# FOXM1 as a candidate dosage rheostat and state selector in breast cancer: linking multilevel regulation to adaptive plasticity and therapy resistance

**DOI:** 10.3389/fonc.2026.1979150

**Published:** 2026-09-28

**Authors:** Ghaleb Oriquat, Hani Moslem Ahmed, Jasur Rizaev, Mamura Kucharova, Djamila Polatova, Mrtda Adnan Mhysn, Navin Kumar Tailor

**Affiliations:** 1 Faculty of Allied Medical Sciences, Hourani Center for Applied Scientific Research, Al-Ahliyya Amman University, Amman, Jordan; 2 College of Pharmacy, Alnoor University, Nineveh, Iraq; 3 Department of Public health and Healthcare management, Rector, Samarkand State Medical University, Samarkand, Uzbekistan; 4 Department of Morphological Sciences, Termez University of Economics and Service, Termez, Uzbekistan; 5 Scientific-Practical Medical Center for Pediatric Oncology, Hematology and Immunology, Tashkent, Uzbekistan; 6 Department of Pharmacy, College of Pharmacy, The Islamic University, Najaf, Iraq; 7 University Institute of Pharma Sciences, Chandigarh University, Mohali, Punjab, India

**Keywords:** adaptive plasticity, breast cancer, FoxM1, therapy resistance, transcriptional dosage

## Abstract

FOXM1, a forkhead-box transcription factor, is consistently overexpressed in breast cancer, particularly in the basal-like and triple-negative subtypes, in which high expression predicts poor survival. We propose, as a falsifiable hypothesis rather than an established mechanism, that FOXM1 may function as a dosage-integrating rheostat, in which convergent genomic, transcriptional, epitranscriptomic, translational, and post-translational inputs would determine discrete output states involving mitotic fidelity, chromosomal instability, metabolic rewiring, immune evasion, and stem-like plasticity. We further hypothesized that therapies directed elsewhere in the network are frequently buffered by the persistence or reactivation of FOXM1-associated outputs, while noting that resistance to direct FOXM1 inhibition proceeds instead through reversal of that output, which bounds the hypothesis rather than supporting it. Accordingly, we classified FOXM1-directed strategies, including direct inhibitors, degraders, condensate disruptors, upstream suppressors, and targeted delivery platforms, by their mechanism of engagement rather than by their chemical class. Existing evidence remains largely correlative and bulk-derived: available perturbation studies are categorical rather than graded, no study has titrated FOXM1 through intermediate levels or measured its outputs bidirectionally, and the few single-cell, time-resolved datasets resolve one output at a time. We identified these gaps, specified the components of an isoform-, localisation- and activation-resolved FOXM1 activity metric that would define dose in measurable terms, and proposed experiments to test threshold-dependent activation, discrete single-cell states, and history-dependent switching, grounding the last of these in the material properties and physical ageing of FOXM1 condensates rather than in an equilibrium phase boundary alone in order to distinguish dosage-dependent state selection from regulatory hub behavior.

## 1 Introduction

The forkhead box (FOX) transcription factor FOXM1 is a core regulator of the mammalian cell division cycle, driving the G1/S and G2/M transitions and the transcriptional program that ensures orderly mitosis and faithful chromosome segregation (1–3). Genetic deletion of FOXM1 results in embryonic lethality (4). FOXM1 has been implicated as an oncogene that regulates multiple aspects of oncogenesis, including tumorigenesis (5–7), metastasis (8, 9), angiogenesis (10, 11), epithelial-to-mesenchymal transition (EMT) (12, 13), tumor immunity (14, 15), and therapy resistance (16–18).

Breast cancer is among the most frequently diagnosed malignancies worldwide and occurs predominantly in women. The marked biological and molecular diversity of this disease complicates the development of precise and effective therapeutic strategies. Advances in molecular profiling have resolved breast cancer into intrinsic transcriptional subtypes: luminal A, luminal B, HER2-enriched, and basal-like, which are approximated in routine practice by immunohistochemical surrogates based on estrogen- and progesterone receptor status, HER2, and Ki-67 (19). Triple-negative breast cancer (TNBC), defined by the absence of estrogen receptor, progesterone receptor, and HER2 expression, is a clinicopathological category that overlaps substantially, though incompletely, with the basal-like intrinsic subtype. This distinction is material to the present synthesis, because FOXM1 abundance and dependence are reported to be greatest in basal-like/TNBC disease, and the two designations are not interchangeable. This heterogeneity also limits what any single-gene marker can be expected to capture. Prognostic associations identified in pooled breast cancer cohorts need not be uniform across molecular subgroups, motivating approaches such as network-fusion methods that seek prognostic biomarkers while accounting for heterogeneous subgroup structure (20). FOXM1 illustrates this limitation: its expression has been associated with breast cancer prognosis (21), while FOXM1 expression is particularly prominent in basal-like and triple-negative disease (22). Moreover, the FOXM1 cistrome predicts metastatic outcome more accurately than FOXM1 expression level or the tumor proliferation index (23), indicating that functional activity may provide information beyond transcript abundance. Heterogeneity therefore represents both a biological source of variation in FOXM1-associated states across breast cancer subtypes and a methodological constraint when prognostic associations are inferred from pooled cohorts. In breast cancer specifically, FOXM1 sits at the convergence of several defining disease processes. Its expression is highest in basal-like and TNBC, where frequent TP53 loss relieves a key restraint on FOXM1, and its overexpression accompanies proliferation, invasion, chemoresistance, and poor survival, marking it as an emerging therapeutic target (24). In estrogen receptor-α (ERα)-positive disease, FOXM1 is a direct transcriptional target of ERα and a principal effector of estrogen-driven proliferation; antiestrogens repress FOXM1 in endocrine-sensitive but not resistant cells (25), and the two factors co-occupy overlapping genomic binding sites (26). Sustained or reactivated FOXM1, acting downstream of 14-3-3ζ signaling and related inputs, drives acquired resistance to tamoxifen and fulvestrant while expanding a stem-like, invasive cell population, coupling FOXM1 activity to both endocrine failure and disease progression (27, 28). Consistent with this, the genome-wide FOXM1 transcriptional program predicts metastatic outcome in breast cancer more accurately than FOXM1 expression levels alone (23). Metabolic reprogramming is one of the co-regulated outputs through which FOXM1 may shape the breast cancer state described above. Altered cancer metabolism supports biosynthetic demands, redox homeostasis, and survival under therapeutic stress (29). In breast cancer, FOXM1 activity has been associated with glycolytic, amino-acid/glutamine-handling, and cholesterol-biosynthetic programs, while FOXM1 abundance is also subject to nutrient-sensitive post-translational regulation. Thus, metabolism may both influence FOXM1 and be influenced by it. Whether these associations constitute a coordinated, FOXM1-dose-dependent metabolic program or reflect subtype- and model-specific effects remains unresolved.

Yet, these advances have accumulated largely in isolation. FOXM1 in breast cancer has typically been studied one pathway and one phenotype at a time, so that its regulation and its consequences are documented as a series of separate mechanisms rather than as a single, integrated system. Two properties are consequently under-addressed. First, the abundance and activity of FOXM1 are set at several distinct regulatory levels, genomic and transcriptional, epitranscriptomic and translational, and post-translational, yet these are seldom considered together as determinants of one functional output. Second, that output is not a single phenotype but several co-regulated programs, mitotic progression and chromosomal instability, metabolic reprogramming, immune evasion, and stem-like plasticity, that are frequently reactivated together under therapeutic pressure. No current synthesis connects how FOXM1 “dose” is established to how it is translated into these co-regulated programs, and the single-cell, dose-resolved data required to test whether those programs constitute discrete cellular states remain scarce.

## 2 FOXM1: gene structure, isoforms, and domain organization

The human FOXM1 gene is located on chromosome 12p13.33 and is organized into eight constitutively expressed exons, designated I–VIII, together with two alternatively spliced cassette exons, Va and VIIa (30–32). Inclusion of exon Va introduces a 15-amino-acid sequence into the forkhead DNA-binding domain (DBD), representing a distinctive structural feature of FOXM1 within the FOX family, whereas exon VIIa adds a 38-amino-acid segment to the C-terminal transactivation domain (TAD) (30, 32, 33). The classical splicing pattern involving these two exons generates three extensively studied isoforms: FOXM1a, which retains both Va and VIIa; FOXM1b, which excludes both alternative exons; and FOXM1c, which contains Va but lacks VIIa (32–34). Although FOXM1a retains the ability to associate with DNA, it exhibits little or no transcriptional activity, whereas FOXM1b and FOXM1c are transcriptionally active isoforms (30, 32, 35–37). FOXM1a, FOXM1b, and FOXM1c comprise 801, 748, and 763 amino acids, respectively, with FOXM1b and FOXM1c sharing approximately 98% amino acid sequence identity (**Figure 1**) (34). High sequence identity does not imply functional equivalence. FOXM1b displayed greater transforming ability than FOXM1c in soft-agar assay, whereas the transactivating activity of FOXM1c, but not that of FOXM1b, was responsive to RAF/MEK/MAPK activation, a difference attributable to an ERK1/2 target sequence carried within exon Va (38). The two transcriptionally competent isoforms are therefore distinguishable both in potency and in the upstream signals to which they respond, a distinction developed in Section 3.2 as a component of functional FOXM1 dose. A fourth splice variant, FOXM1d, has been identified. This 786-amino-acid isoform lacks exon Va but retains exon VIIa, localizes predominantly to the cytoplasm, and exerts a non-transcriptional function through direct interaction with ROCK2 and, to a lesser extent, with ROCK1, thereby promoting ROCK signaling (36).

**Figure 1:**
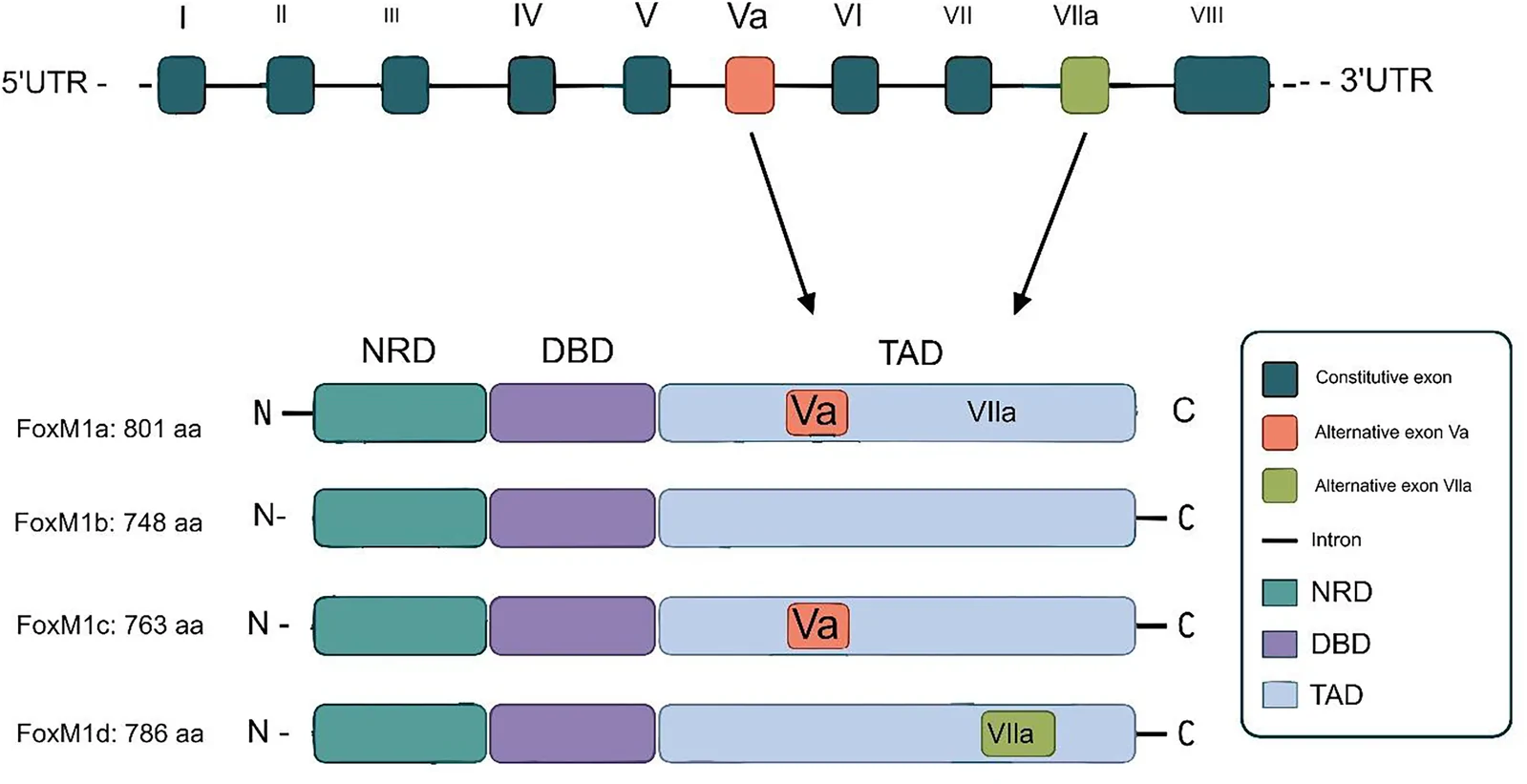
FOXM1 gene organization, alternative splicing, and isoform-specific protein structures. Schematic illustration of the human FOXM1 gene, showing eight constitutive exons (I–VIII) and two alternatively spliced exons (Va and VIIa). Alternative inclusion of these exons generates distinct FOXM1 isoforms: FOXM1a (801 aa) containing both Va and VIIa, FOXM1b (748 aa) lacking both, FOXM1c (763 aa) containing Va only, and FOXM1d (786 aa) retaining VIIa but lacking Va. FOXM1 proteins comprise the N-terminal negative regulatory domain (NRD), central DNA-binding domain (DBD), and C-terminal transactivation domain (TAD), with alternative exon incorporation contributing to their isoform-specific functional properties. FOXM1d, which is predominantly cytoplasmic and non-transcriptional, is shown here for completeness but is not carried forward into the splicing layer of **Figure 2** (Section 2) [Redrawn from Zhang et al. (203)].

**Figure 2:**
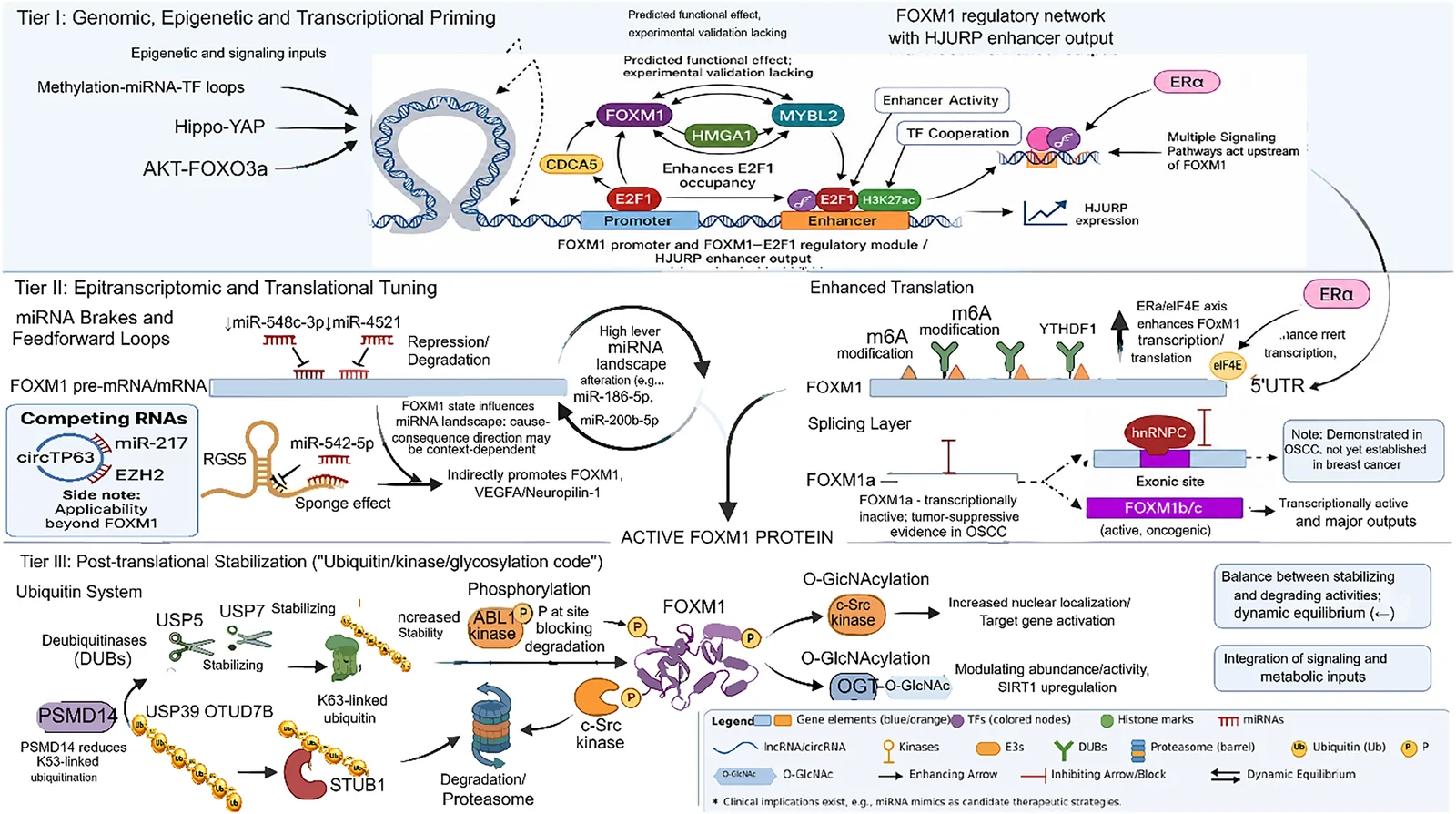
Multilevel regulation of the FOXM1 setpoint. Tier I establishes the transcriptional baseline through enhancer activity, transcription factor cooperation, sequence variation, and epigenetic/signaling inputs. Tier II tunes FOXM1 through miRNA and competing-RNA networks, m6A/YTHDF1- and eIF4E-dependent translation, and isoform-specific splicing; the splicing layer depicts FOXM1a, FOXM1b and FOXM1c only, because FOXM1d is cytoplasmic and non-transcriptional and therefore does not contribute to nuclear transcriptional dose, entering the model instead through the nuclear-fraction term of **Figure 3** (Section 2). Tier III adjusts protein stability and activity through ubiquitination, phosphorylation, and O-GlcNAcylation; USP39 is not shown here, being a catalytically inactive pseudo-DUB whose likely point of action lies in the Tier II splicing layer (Section 6). Together, these layers support a distributed dosage-setting model, although their hierarchy, context dependence, cross-talk, and quantitative contributions remain unresolved.

**Figure 3:**
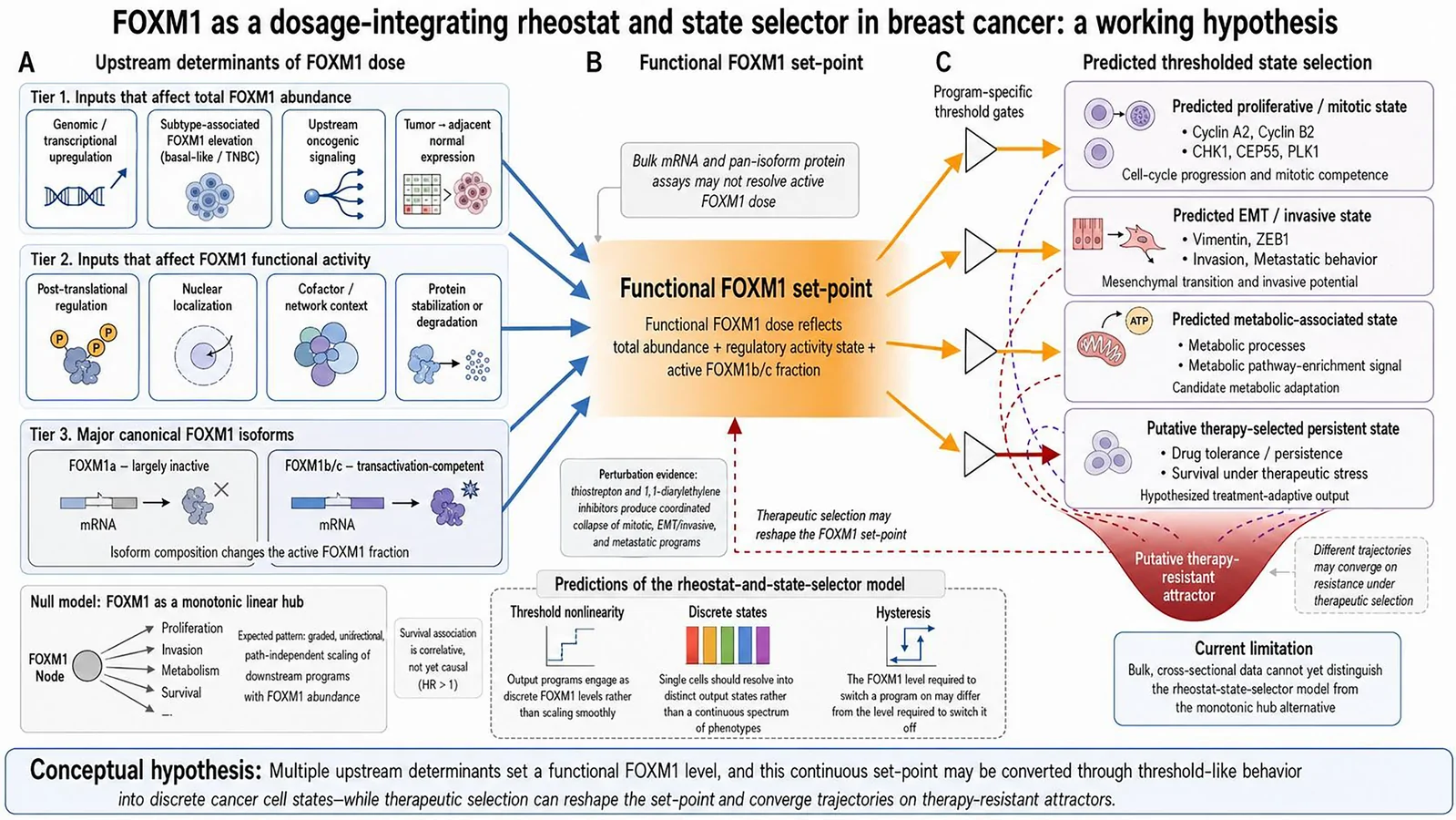
Multilevel regulation of the FOXM1 setpoint. The regulatory landscape is organized into three tiers: Tier I (Genomic, Epigenetic and Transcriptional Priming), which establishes the transcriptional baseline through enhancer activity, transcription factor cooperation, sequence variation, and epigenetic/signaling inputs; Tier II (Epitranscriptomic and Translational Tuning), which modulates FOXM1 expression through miRNA and competing-RNA networks, m6A/YTHDF1- and eIF4E-dependent translation, and isoform-specific splicing; and Tier III (Post-translational Stabilization; “Ubiquitin/kinase/glycosylation code”), which regulates FOXM1 protein stability and activity through ubiquitination, phosphorylation, and O-GlcNAcylation mechanisms. The splicing layer depicts FOXM1a, FOXM1b, and FOXM1c only, as FOXM1d is cytoplasmic and non-transcriptional and therefore does not contribute to nuclear transcriptional dose; instead, it is incorporated into the model through the nuclear-fraction term in **Figure 2** (Section 2). USP39 is not shown in Tier III because it is a catalytically inactive pseudo-DUB whose proposed role is primarily associated with the Tier II splicing layer (Section 6). Together, these regulatory layers support a distributed dosage-setting model, although their hierarchy, context dependence, cross-talk, and quantitative contributions remain unresolved.

A scope convention follows from this distinction and applies to the figures. FOXM1d is described here to complete the account of FOXM1 alternative splicing, but it is not included in the nuclear transcriptional layer of **Figure 2**. In the study that identified this isoform, FOXM1d was found to reside predominantly in the cytoplasm and to promote colorectal cancer EMT and metastasis through direct interaction with and activation of ROCK1/2, distinguishing it from the canonical transcription-related functions of FOXM1b/c (36). The same study quantified FOXM1d primarily in colorectal cancer models, with additional measurements in lung cancer cell lines, but did not examine breast cancer models (36). Accordingly, **Figure 2** restricts its transcriptional layer to FOXM1a/b/c, while FOXM1d is represented separately as a predominantly cytoplasmic, non-canonical FOXM1 isoform. This is a framework convention rather than evidence that FOXM1d has no transcription-related effects, and it should be reconsidered if breast-specific evidence establishes a substantial role for FOXM1d.

FOXM1 comprises an N-terminal negative regulatory domain (NRD), a centrally positioned and evolutionarily conserved forkhead DBD, and a C-terminal TAD (36, 39–41). Structural studies have shown that the FOXM1 DBD can recognize forkhead-related DNA elements containing the TAAACA motif; however, its genomic occupancy is not restricted to this canonical sequence (41–43). Genome-wide analyses have indicated that FOXM1 can directly recognize certain non-consensus DNA elements and can also be indirectly recruited to cell cycle-regulated promoters through interactions with B-MYB/MYBL2 and the MuvB complex (26, 42, 44, 45). Through these direct and partner-assisted binding mechanisms, FOXM1 coordinates the temporal expression of genes required for progression through the late stages of the cell cycle (42, 44, 45).

FOXM1 TAD promotes target gene transcription by recruiting the transcriptional coactivators CBP and p300 in a phosphorylation-dependent manner (46, 47). In the inactive state, intramolecular association between the NRD and TAD restricts access of these coactivators and maintains FOXM1 in an autorepressed conformation (47, 48). Cell cycle-dependent phosphorylation by cyclin–CDK complexes disrupts this inhibitory interaction, enhances coactivator recruitment, and progressively increases FOXM1 transcriptional activity during the G2/M transition (39, 46–48). FOXM1 activity can also be modified by additional kinase-dependent signals in specific biological settings; for example, in luminal B-like breast cancer models, c-Src phosphorylates FOXM1 at two tyrosine residues, thereby enhancing its nuclear localization and target gene expression (49).

Beyond its established role in cell cycle control, FOXM1 contributes to cellular proliferation, differentiation, tissue homeostasis, repair, and the maintenance of stem cell properties, with effects that vary according to cell type and biological context (50). These functions are mediated partly through the transcriptional regulation of downstream genes involved in mitotic progression and chromatin organization, including CCNB1, CCNB2, PLK1 and ECT2 (26, 34, 42–45). FOXM1 also engages in functional crosstalk with several major signaling networks, including Wnt/β-catenin, TGF-β/SMAD, and STAT3 pathways, thereby extending its influence beyond the direct transcriptional regulation of canonical cell-cycle genes (34, 51, 52).

Evidence from non-malignant disease models further illustrates the context-dependent significance of FOXM1 expression. A rare potentially pathogenic FOXM1 variant was identified in a family affected by maturity-onset diabetes of the young, suggesting that impaired FOXM1 function may contribute to selected forms of monogenic diabetes rather than diabetes in general (53). In human diabetic wounds, disruption of FOXM1-dependent immune programs has been associated with defective recruitment, activation, and survival of neutrophils and macrophages, thereby contributing to impaired tissue repair (54). In experimental liver models, hepatocyte-specific FOXM1 overexpression increases CCL2 secretion and promotes macrophage recruitment, hepatic inflammation, tissue injury, and fibrosis (55). FOXM1 also functions downstream of NOTCH signaling to support mitochondrial biogenesis during the generation of human stem cell memory-like CAR-T cells (56). In addition, bioinformatic analyses of circulating miRNAs associated with myasthenia gravis have identified FOXM1 among several forkhead-family transcription factors with potential binding sites in the regulatory regions of disease-associated miRNAs, although a direct disease-specific regulatory role for FOXM1 remains to be experimentally established (57). Collectively, these findings indicate that alterations in FOXM1 expression, splicing, subcellular localization, or activation may influence a broad spectrum of physiological and pathological processes in a highly cell- and context-dependent manner (50, 53–57).

## 3 FOXM1 as a candidate systems-level rheostat: framing the problem

In breast cancer, the forkhead transcription factor FOXM1 occupies a position that is simultaneously clinical and conceptual. Expression surveys consistently rank it among the most strongly upregulated regulators, rising sharply in tumors relative to adjacent normal tissue and reaching its highest levels in the basal-like and triple-negative subtypes (21, 22). This is more than a descriptive correlation: elevated FOXM1 is associated with shorter patient survival, and network-based analyses recurrently recover it as a hub rather than a peripheral node, placing it at a convergence point within the breast-cancer gene-regulatory architecture (21, 22).

What separates FOXM1 from an ordinary proliferation marker is the breadth of programs that contract when its activity is lowered. Two structurally unrelated interventions, the thiazole antibiotic thiostrepton and a class of 1,1-diarylethylene inhibitors, converge on the same outcome: simultaneous loss of mitotic and cell-cycle genes (cyclin A2, cyclin B2, CHK1, CEP55, PLK1), downregulation of epithelial–mesenchymal markers such as vimentin and ZEB1, and suppression of invasion and metastasis in both culture and xenograft models (22, 58). One qualification attaches to this convergence at the outset. Thiostrepton interacts directly with FOXM1 (59), but thiostrepton and the related thiazole antibiotic siomycin A also inhibit the proteasome, while proteasome inhibition suppresses FOXM1 as a general property (60). Effects obtained with thiostrepton are therefore not attributable to FOXM1 engagement alone (Section 12.1). The convergence described here is consequently carried principally by the 1,1-diarylethylene series (58) and by the concordant genetic evidence cited throughout the sections that follow, with the thiostrepton arm providing supporting rather than independent confirmation. Pathway-enrichment analysis additionally links FOXM1 to metabolic processes (22). This convergence is the observation that motivates the framework, though its evidential reach must be stated precisely. A coordinated, multi-program collapse following removal of a single factor is difficult to reconcile with a linear effector acting through any one pathway, and instead identifies FOXM1 as a hub or integrator whose level bears on several outputs at once (22, 58). It does not, by itself, distinguish a thresholded rheostat from a linear multi-output hub, a distinction developed below. Two cautions qualify the picture. The prognostic relationship remains correlative (hazard ratio > 1) rather than demonstrably causal (21), and FOXM1 “activity” is inferred almost entirely from bulk expression, leaving its functional dose without a quantitative definition (21, 22) (**Figure 3**). This gap has a specific molecular basis that recurs throughout the argument below. FOXM1 is expressed as three splice isoforms, FOXM1a, FOXM1b and FOXM1c, that differ sharply in transcriptional activity: FOXM1b and FOXM1c are transactivation-competent, whereas alternative-exon inclusion in FOXM1a disrupts its activation domain and renders it largely inactive (35, 38). Bulk mRNA measurements and pan-isoform antibodies therefore conflate transcriptionally competent and incompetent species. Notably, the inactive FOXM1a isoform is generally expressed at low or undetectable levels in breast tumors and cancer cell lines, so the compositional variable most likely to shape functional output is not simply the active-to-inactive ratio but the relative abundance of the two active isoforms, FOXM1b and FOXM1c, which differ in transforming potency and in their responsiveness to RAF/MEK/ERK signaling (38). On either reading, two tumors with identical total FOXM1 may differ in functional output according to isoform composition, a compositional dimension of “dose” that current bulk data cannot resolve. In the terms developed here, “dose” is consequently not merely how much FOXM1 is present but how much active FOXM1, a compositional as well as quantitative variable that current bulk data cannot resolve. These limitations nonetheless frame the organizing thesis developed below, which proposes that FOXM1 may behave as a dosage-integrating rheostat and state selector rather than as a conventional single-pathway oncogene. Framed this way, the thesis carries an explicit and falsifiable burden: it must be distinguished from the simpler null in which FOXM1 is a highly connected but linear hub, where every downstream program scales smoothly and monotonically with FOXM1 abundance. The rheostat-and-state-selector model is best understood as analog-to-digital: a continuous set-point (the “rheostat”) would be converted through thresholds into discrete output states (the “state selector”), and it is this hypothesized thresholded conversion, not smooth dimming, that would separate the model from a monotonic hub. Three qualitative predictions follow that the hub null does not make. First, threshold nonlinearity: output programs should engage at discrete FOXM1 levels rather than rising continuously with dose. Second, discreteness and partial mutual exclusivity: at single-cell resolution, outputs should resolve into distinct states rather than a smooth continuum. Third, hysteresis: the FOXM1 level at which a program switches on should differ from that at which it switches off, the signature of a bistable switch. The monotonic-hub alternative predicts none of these, instead expecting graded, unimodal, path-independent scaling of all outputs with dose. It is essential to be explicit about what does and does not adjudicate between them. Both models place FOXM1 at a convergence point: a single factor integrates many upstream inputs, and its removal collapses several downstream programs in parallel. Convergence of this kind, whether of upstream regulators onto FOXM1 or of mechanistically distinct perturbations onto a shared FOXM1-associated output, is therefore a necessary but not a sufficient feature of the rheostat-and-state-selector model; it is equally expected under the linear-hub null and is not, by itself, evidence for state selection. Only the three signatures above, threshold nonlinearity, single-cell multimodality with partial mutual exclusivity, and hysteresis, distinguish the two. Accordingly, the recurring convergences documented in the sections that follow should be read as motivating the hypothesis and establishing FOXM1 as a hub, not as confirming dose-dependent state selection. Critically, the bulk, cross-sectional data on which the present synthesis rests cannot yet discriminate between these alternatives; the distinction is therefore advanced here as a testable prediction rather than an established property, with the adjudicating experiments specified in the concluding sections. A reading convention follows from this breast-cancer framing. Because the synthesis is specific to breast cancer, any finding drawn from a non-breast system is marked at first mention with an italic tag naming that system, *(non-breast: system)*, so that the breast-specific portion of the scaffold can be separated at a glance from cross-tissue material, which is cited only where it supplies mechanistic plausibility or a transferable logic, never as direct breast-cancer evidence. **Tables 1**–**3** contain only breast-derived entries. A second convention concerns the vocabulary of the model itself. The terms “rheostat,” “state selector,” “analog-to-digital,” “threshold,” “dosage-dependent state selection” and “shared attractor” name properties proposed in this article that have not been demonstrated for FOXM1 in breast cancer, nor, so far as we are aware, in any system. They are retained rather than softened because they specify the model precisely enough to be falsified; a vaguer vocabulary would be harder to test, not more cautious. Their retention should not, however, be read as assertion. Throughout the text they are therefore used only in the modal register, as what the model proposes, predicts or would require, while the indicative is reserved for reported observations. Section headings follow the same convention, using “candidate output state” and “candidate shared attractor” where the framework’s vocabulary is unavoidable. The intention is that a reader should be able to determine from the grammar of any sentence in this article whether it reports a finding or states a prediction, and any place where that distinction is not recoverable is an error on our part rather than a claim.

**Table 1:** Post-translational regulators of FOXM1 in breast cancer (input tier III).

Enzyme/regulator	PTM type	Effect on FOXM1 (stability vs. activity)	Direction	Ref.
*USP5*	Ubiquitin (DUB)	Removes ubiquitin and blocks proteasomal degradation, increasing FOXM1 protein levels; USP5 knockdown accelerates FOXM1 degradation.	Stabilizes ↑	(79)
*USP7*	Ubiquitin (DUB)	Deubiquitinates and stabilizes FOXM1 in TNBC; USP7 inhibition/degradation lowers FOXM1 and suppresses growth p53-independently.	Stabilizes ↑	(80)
*USP39*	Ubiquitin-related, non-catalytic; USP39 is a spliceosomal factor required for stable recruitment of the U4/U6.U5 tri-snRNP to the pre-spliceosome and mature spliceosome assembly. Although it contains a USP-like domain, the canonical catalytic residues are not conserved, and no ubiquitin C-terminal hydrolase/deubiquitinating activity has been detected experimentally (111–114)	USP39 overexpression is associated with lower FOXM1 ubiquitination, increased FOXM1 protein and increased target-gene transcription (Cdc25b, PLK1) (81). Because USP39 cannot function as an isopeptidase, the mechanism is not direct deubiquitination; adaptor or scaffolding action on an active DUB, or a splicing-dependent indirect route, are the available alternatives (Section 6)	Stabilizes ↑/activity ↑ *(mechanism unresolved)*	(81, 111–114)
*OTUD7B*	Ubiquitin (DUB, OTU family)	Binds FOXM1 and reduces its polyubiquitylation; knockdown lowers FOXM1 and curbs proliferation and stemness.	Stabilizes ↑	(82)
*PSMD14*	Ubiquitin (proteasome-associated DUB)	It reduces K63-linked ubiquitination of FOXM1 and co-activates PI3K/AKT/mTOR.	Stabilizes ↑	(83)
*STUB1*	Ubiquitin (E3 ligase)	Targets FOXM1 for ubiquitin-mediated degradation, thereby repressing FOXM1-driven transcription (e.g. TOP2A).	Destabilizes ↓	(74)
*ABL1*	Phosphorylation (Tyr)	Phosphorylates FOXM1 at pY575, blocking APC/C–CDH1 recognition and protecting it from degradation; loss of phosphorylation shortens half-life.	Stabilizes ↑	(75)
*c-Src*	Phosphorylation (Tyr)	It phosphorylates two tyrosine residues to drive FOXM1 nuclear localization and target gene expression within a positive feedback loop.	Activity ↑	(49)
O-GlcNAc – SIRT1 axis	O-GlcNAcylation	Lowering O-GlcNAc elevated SIRT1, which promotes MEK/ERK-dependent proteasomal degradation of FOXM1; O-GlcNAc thus supports FOXM1.	Stabilizes ↑ (loss ↓)	(84)
OGT/O-GlcNAc	O-GlcNAcylation	Reducing O-GlcNAc (OGT depletion) decreases FOXM1 expression and activity and its downstream targets (e.g., Skp2).	Expression/activity ↑	(76)

USP39 is retained in this table because the reported effect on FOXM1 abundance is a genuine observation, but it is annotated as a pseudo-DUB and is not counted among the catalytically active deubiquitinases converging on FOXM1. Its most likely point of action lies in Tier II rather than Tier III; see Section 6. All entries are derived from breast cancer systems. In the Direction column, ↑ denotes an increase and ↓ a decrease in the stated FOXM1 property (protein stability/abundance, or transcriptional activity) caused by the listed regulator; "Stabilizes ↑" therefore means the regulator raises FOXM1 protein levels and "Destabilizes ↓" that it lowers them, while "Activity ↑" refers to increased FOXM1 activity without a demonstrated change in abundance. "Stabilizes ↑ (loss ↓)" indicates that loss of the modification lowers FOXM1. The arrows denote the direction of effect only and carry no magnitude.

**Table 2:** Subtype-stratified comparison of FOXM1 regulation, output and resistance in breast cancer.

Subtype of model	FOXM1 regulatory mechanism	Downstream pathway/output	Resistance phenotype	Experimental model	Evidence grade
ER+ (luminal)	Direct ERα transactivation via an ERE in the FOXM1 promoter	Estrogen-driven proliferation; mitotic gene program	Tamoxifen resistance; FOXM1 no longer repressed by 4-OHT in resistant cells	MCF-7 and tamoxifen-resistant MCF-7 derivatives; FOXM1 silencing and ectopic expression	B (25)
ER+ (luminal)	RGC-32/PI3K amplification of ERα activity (154); AREG–EGFR–PI3K/AKT/mTOR retention of ERα under aromatase-inhibitor pressure (155)	Elevated FOXM1 (and SGK3 (155)); estrogen-independent ERα output	Tamoxifen resistance (154); aromatase-inhibitor resistance (155)	Tamoxifen-resistant and AI-resistant ER+ lines; knockdown of the upstream regulator, not of FOXM1	C (154, 155)
ER+/HER2− (clinical)	Switch from ERα-dependent to HER2-dependent, NF-κB-supported FOXM1 expression	FOXM1 maintenance; tumor cell survival	Endocrine-therapy escape; NF-κB inhibition blocks HER2 and FOXM1 and induces apoptosis	Circulating tumor cells from metastatic ER+/HER2− patients. *HER2-non-amplified disease*	C (118)
ER+ (luminal)	Persistence of FOXM1 expression after PI3Kα inhibition; no FOXM1 perturbation reported in the work cited here	Downstream metabolic reprogramming (e.g. LDH)	PI3Kα-inhibitor resistance; FOXM1 persistence proposed as a response biomarker	ER+ models; serial hyperpolarised ¹³C imaging *in vivo*	D (66)
ER+ (luminal)	FOXO3-dependent downregulation of FOXM1 required for AKT-inhibitor efficacy	Proliferation	Capivasertib resistance on FOXO3 loss or sustained FOXM1; reversed by FOXM1 downregulation	PIK3CA- and PIK3CA/PTEN-altered ER+ models	B (156)
Luminal B-like	c-Src tyrosine phosphorylation → enhanced nuclear localisation and target-gene activation, within a positive feedback loop	Tumor cell proliferation; breast cancer progression	—	Luminal B-like mouse mammary tumor models, *in vivo*	B (49)
HER2-amplified	No study identified in this reference set in which FOXM1 is perturbed in a HER2-amplified breast cancer model	—	—	—	Gap (see Section 7)
TNBC/basal-like	Transcriptional priming: FOXM1/E2F1/H3K27ac enhancer occupancy (97); methylation–miRNA–TF feed-forward loops placing FOXM1 among principal TFs (98)	HJURP transcription (97); mitotic gene program	—	TNBC lines with enhancer interference (97); integrative bioinformatic analysis of basal-like tumors (98)	C (97); D (98)
TNBC/basal-like	FOXM1 knockdown; direct promoter binding	eEF2K (119); KIF23 → Wnt/β-catenin (121)	—	TNBC lines with xenograft confirmation	B (119, 121)
TNBC/basal-like	FOXM1 knockdown or silencing; YTHDC1/m6A-dependent modulation (108); FOXM1 with FUS (123)	SQLE induction, reported as glycolytic (125) but reassigned here to the cholesterol arm (Section 8); CENPA-dependent glycolysis (108); SLC7A5/LAT1 glutamine handling (123)	Anoikis suppression (125)	TNBC lines; rescue by re-expression of the effector	C (108, 123, 125)
TNBC/basal-like	Tumor-intrinsic FOXM1 CRISPR/Cas9 knockout	DNMT1–UHRF1 → STING silencing → CHOP-dependent ULBP1 → NKG2D-mediated NK and T cell cytotoxicity	Reduced immunotherapy responsiveness; impaired immune memory	TNBC lines; syngeneic mouse models; single-cell RNA-seq; patient survival and ICI-response correlation	A (143). *Single study, not independently replicated*
TNBC/basal-like	FOXM1 siRNA (lipid nanoparticle) (140); exosomal transfer of FOXM1 to recipient macrophages (141)	KLF15 → M1 repolarization on FOXM1 loss (140); IDO1 transcription and ferroptosis suppression (141); KIF20A (142)	Reduced immune-checkpoint-inhibitor efficacy when FOXM1 is high (140)	TNBC lines; macrophage co-culture; *in vivo* ICI combination (140)	B (140–142)
TNBC (AR-low)	None. Correlative expression analysis only; FOXM1 not perturbed	15-gene mitotic kinesin, centromeric and proteolysis signature; overlap with chromosomal-instability gene sets	Shorter relapse-free survival	TCGA, METABRIC, UALCAN, KM Plotter and TIMER analyses; no wet-laboratory experiment	D (122)
TNBC/basal-like	FOXM1 silencing (61); FOXM1 inhibition (thiostrepton) with ACSL4 targeting (62); FDI-6 or FOXM1 shRNA (162); BET inhibition disrupting FOXM1–MYC (163)	DNA-repair gene expression with NF-κB (61); stem-like CD24−/CD44+ state (62); FOXM1/Exo1 and HR competence (162); FOXM1–MYC co-occupancy at HR loci (163)	Doxorubicin, radiation and PARP-inhibitor resistance; re-sensitization on FOXM1 suppression	TNBC lines with xenografts; BRCA-proficient models	B (61, 62, 162, 163)
TNBC/basal-like	PI3K inhibition (BKM120) lowering FOXM1 and Exo1; FOXM1 not directly perturbed	PI3K/Akt/FOXM1/Exo1 axis; homologous-recombination capacity	Olaparib resistance in BRCA-proficient cells; conversion to an HR-deficient, olaparib-sensitive state	BRCA-proficient TNBC lines	C (161)
ER+ vs TNBC *(discordant)*	FOXM1 repression (siRNA, FDI-6, RCM-1); FOXM1 overexpression	BMF de-repression; death in mitosis versus mitotic slippage, scored per cell by time-lapse imaging	Antimitotic-drug tolerance	Human dermal fibroblasts (discovery); MCF-7 FOXM1-dependent; MDA-MB-231 BMF repression FOXM1-independent	B (63). *Subtype-discordant*
ER+ and TNBC	Inducible, isoform-specific FOXM1b or FOXM1c expression; FOXM1 knockdown	Mitotic duration, mitotic error rate and proliferative fitness under MAD2-driven chromosomal instability	Tolerance of, and fitness recovery from, chromosomal instability	MCF-7, CAL51, MDA-MB-231 with inducible MAD2; single-cell time-lapse imaging	B (64)
Cross-subtype	Acquired resistance to FOXM1 inhibitors themselves	Ferroptosis and apoptosis suppression with elevated CSC markers (16); genome rewiring to HER2/EGFR and inflammatory programs (65); JAK/STAT1–interferon–ISGylation network shared with CDK4/6-inhibitor resistance (165)	Resistance arising after direct FOXM1 inhibition; cross-sensitivity retained, permitting sequential targeting (165)	Breast cancer lines with derived resistant sublines	B (16, 65, 165)
Non-breast *(gastric, colorectal)*	AKT-dependent FOXO3a phosphorylation → FOXM1 downregulation on orlistat treatment	PD-L1 transcription; interferon-stimulated genes; MHC-I	Modulates anti-CTLA-4 immunotherapy efficacy	AGS (gastric) and HCT116 (colorectal) cells. No breast cancer model	Ungraded (144). *Tagged non-breast; cited for transferable mechanism only*

Evidence grades. A — direct genetic perturbation of FOXM1 in a breast cancer model, with *in vivo* confirmation and independent patient-sample validation. B — direct genetic perturbation of FOXM1 in a breast cancer model, without both of the above. C — modulation by a pharmacological agent, or by genetic perturbation of an upstream regulator only, where the FOXM1-specific contribution has not been isolated. D — association only: expression correlation, occupancy mapping, or database and bioinformatic analysis without perturbation of FOXM1. Grades reflect the study design as reported in the sections of this review that cite each study, and describe the strength of the evidence rather than the plausibility of the conclusion. No entry in this table meets the dose-resolved or multi-output single-cell standard set out in Section 3.1 and (**Box 1**). Em dashes denote fields not addressed by the cited work. The SQLE entry is graded C rather than B because, although FOXM1 was genetically perturbed, the glycolytic output was indexed by extracellular acidification and metabolite pool sizes rather than by flux, and no mechanism linking a cholesterogenic enzyme to glycolysis was identified or tested (Section 8).

**Table 3:** FOXM1-associated output across therapy-resistance contexts in breast cancer: evidence bearing on the candidate shared-attractor model.

Therapy class	How FOXM1 sustains resistance	Re-sensitization strategy reported	Ref.
A. Endocrine and PI3K/AKT therapy resistance (ER+ breast cancer)			
Tamoxifen/anti-estrogen (ERα)	FOXM1 is a direct ERα transcriptional target via an ERE in its promoter; ectopic FOXM1 overrides anti-estrogen–mediated cell cycle arrest, and it is no longer repressed by OHT in resistant cells.	Silencing FOXM1 abolishes estrogen-driven proliferation and reverses acquired tamoxifen resistance	(25)
Tamoxifen (RGC-32–PI3K–ERα)	RGC-32 activates PI3K signaling and enhances ERα activity, thereby elevating FoxM1 levels. Tamoxifen resistance is partly FoxM1-dependent.	RGC-32 knockdown restores tamoxifen sensitivity in resistant cells	(154)
Aromatase inhibitor (AREG–EGFR–PI3K/Akt/mTOR)	Amphiregulin sustains ERα expression and signaling through the PI3K/Akt/mTOR pathway and upregulates FOXM1 (and SGK3), maintaining an estrogen-independent ERα output.	Targeting AREG–ERα crosstalk (AREG is critical for AI-resistant cell viability).	(155)
Endocrine (ERα→HER2 switch, NF-κB)	In circulating tumor cells, FOXM1 expression switches from ERα-dependent to HER2-dependent/ERα-independent, and NF-κB sustains HER2 and FOXM1 under ERα inhibitors.	NF-κB inhibition blocks HER2 and FOXM1 expression and induces cell apoptosis.	(118)
PI3Kα inhibitor	Persistent FOXM1 expression after treatment marks resistance and drives downstream metabolic reprogramming (e.g., LDH).	FOXM1 persistence serves as a response biomarker (detectable by hyperpolarized ¹³C-MRI) to guide the combination design.	(66)
AKT inhibitor (capivasertib)	Efficacy is FOXO3-dependent with FOXM1 downregulation, and FOXO3 loss or long-term AKTi exposure sustains/raises FOXM1 and confers resistance.	Downregulation of FOXM1 reverses capivasertib resistance.	(156)
Tamoxifen (PI3K/p110α)	Loss of N6AMT1 in resistant cells stabilizes p110α (escaping NEDD4L-mediated degradation), raising p-AKT, and sustaining the PI3K/AKT output feeding FOXM1.	N6AMT1 overexpression or the p110α inhibitor A66 restored tamoxifen sensitivity.	(157)
B. DNA-damage, chemotherapy, radiation, PARP, CDK4/6 and FOXM1-inhibitor resistance			
Anthracycline chemotherapy (doxorubicin)	FOXM1 maintains DNA repair gene expression and partners with NF-κB to limit doxorubicin-induced DNA damage.	FOXM1 silencing sensitizes cells to doxorubicin both *in vitro* and in xenografts.	(61)
Radiotherapy	The ACSL4–FOXM1 axis supports radioresistance and a stem-like CD24−/CD44+ phenotype by sustaining the DNA-damage response.	Inhibition of ACSL4 (siRNA/triacsin C) or FOXM1 (thiostrepton) restored radiosensitivity.	(62)
Antimitotic chemotherapy	High FOXM1 represses the pro-apoptotic effector BMF via a BMF intronic cis-regulatory element, limiting death in mitosis during prolonged mitotic arrest. This mechanism operates in MCF-7 but is disrupted in anoikis-resistant MDA-MB-231 cells, in which BMF repression is FOXM1-independent.	FOXM1 repression (siRNA, FDI-6 or RCM-1) upregulates BMF and shifts single-cell fate towards mitotic death; the effect is substantially attenuated in the anoikis-resistant background.	(63)
Anthracycline (doxorubicin, TNBC)	The FOXM1–NF-κB–RAS/MAPK axis sustains pro-survival signaling and DNA repair capacity.	Flavopiridol suppresses FOXM1/NF-κB and RAS/MAPK pathways and lowers repair (↓PARP), sensitizing cells to doxorubicin; FOXM1 silencing recapitulates this.	(158)
Anthracycline (doxorubicin, TNBC)	FOXM1, induced by doxorubicin in addition to RAD51 and survivin, supports DNA repair and survival.	Morin suppresses FOXM1 and attenuates EGFR/STAT3 signaling, sensitizing cells to doxorubicin	(159)
Taxane (docetaxel)	MELK–FoxM1–ABCB1 cascade increases drug efflux to drive docetaxel resistance.	Maslinic acid suppresses MELK and FoxM1, lowers ABCB1, and restores docetaxel sensitivity.	(160)
PARP inhibitor (olaparib)	Olaparib induces adaptive gain of FOXM1 and Exo1 (with repair factors) via the PI3K/Akt/FOXM1/Exo1 pathway, sustaining HR repair in BRCA-proficient cells.	BKM120 (a PI3K inhibitor) downregulated FOXM1/Exo1, inducing HR deficiency and olaparib sensitivity.	(161)
PARP inhibitor (olaparib)	Olaparib induces adaptive upregulation of FOXM1, PARP1/2, BRCA1/2, and Rad51 to activate DNA repair.	FDI-6 (or FOXM1 shRNA) repressed FOXM1 and converted BRCA-proficient TNBC to an olaparib-sensitive, HR-deficient state.	(162)
PARP inhibitor (olaparib)	BRD4-dependent FOXM1–MYC axis co-occupies HR-gene regulatory regions (BRCA1/2, RAD51); sustained FOXM1 silencing triggers compensatory MYC that preserves repair.	BET inhibition (JQ1) or BRD4 degradation (ZBC260) disrupts FOXM1/MYC, induces BRCAness, and synergizes with olaparib.	(163)
FOXM1 inhibitor	Acquired resistance suppresses ferroptosis and apoptosis and elevates cancer stem cell markers.	Low-dose ferroptosis activators (dihydroartemisinin, JKE1674) overcome resistance	(16)
FOXM1 inhibitor	Resistance reverses the FOXM1 network through genome rewiring, activates inflammatory and HER2/EGFR programs, and lowers ERα.	Resistant cells gained approximately 10-fold sensitivity to lapatinib, allowing the targeting of the rewired HER2/EGFR state.	(65)
CDK4/6 inhibitor	FOXM1 and PLK1 were co-regulated and positively correlated within the RB1 pathway under CDK4/6 inhibition.	FOXM1–PLK1 co-regulation within RB1 is proposed as an actionable node (no direct reversal demonstrated).	(164)
FOXM1 inhibitor & CDK4/6 inhibitor	Both resistant states share an elevated JAK/STAT1–interferon–ISGylation network (↑ISG15 and conjugates).	Cross-sensitivity is retained: FOXM1i still inhibits CDK4/6i-resistant cells, and palbociclib/abemaciclib overcomes FOXM1i resistance (sequential targeting). ISGylation knockdown impairs the resistant cells.	(165)
Chemotherapy (doxorubicin, paclitaxel)	The related forkhead factor FOXA1 (not FOXM1) promotes drug resistance and anchorage independence when overexpressed.	FOXA1 knockdown increases doxorubicin and paclitaxel sensitivity — evidence of broader forkhead participation in drug response	(166)

All entries derive from breast cancer systems; the evidence establishes marker convergence and does not address dynamical convergence (Section 11).

Scope and literature search. This article is a narrative, mechanism-focused review rather than a systematic review, and does not follow PRISMA methodology; its aim is conceptual synthesis, not exhaustive enumeration. Literature was identified by searching PubMed/MEDLINE, Scopus, and Web of Science for records published through June 2026, with Google Scholar used for citation tracing, combining the terms “FOXM1,” “FoxM1,” or “forkhead box M1” with “breast cancer,” “triple-negative,” “endocrine resistance,” “chemoresistance,” “cell cycle,” “metabolism,” “immune evasion,” “cancer stem cell,” “phase separation,” and “inhibitor,” “degrader,” or “therapeutic.” Reference lists of retrieved articles were screened for further sources. Priority was given to peer-reviewed primary studies in breast cancer models; findings from non-breast systems were included only where they establish a transferable mechanism or supply mechanistic plausibility and are tagged accordingly in the text. English-language reports were included; conference abstracts were used only to contextualize their corresponding full papers and are not counted as independent evidence. Any cited study carrying an editorial notice bearing on data integrity is flagged and excluded from the synthesis. Where a source report assigns a molecule to a metabolic pathway, that assignment was checked against the enzyme’s established catalytic and regulatory function rather than adopted as stated; where the two diverge, the association is reported and the pathway attribution is withheld pending an identified intervening mechanism. Because sources were selected for mechanistic relevance rather than by predefined systematic criteria, selection bias cannot be excluded, and the review reflects a current, unevenly distributed evidence base rather than a comprehensive tally.

### 3.1 Resolution of the current evidence: what dose-, single-cell- and time-resolved data do and do not exist

Because the distinction between a graded FOXM1 rheostat and a thresholded state selector is empirical, it requires three forms of resolution: FOXM1 dosage, single-cell state, and temporal history. The evidence assembled here is unevenly distributed across these axes.

#### 3.1.1 Dose resolution

A direct test of threshold nonlinearity requires FOXM1 abundance to be varied across several intermediate levels within the same cellular background, with downstream outputs quantified as a function of FOXM1 abundance to estimate dose–response curves and their steepness. Most breast cancer studies instead use categorical FOXM1 depletion, inhibition, or ectopic overexpression (16, 61–64). These designs establish requirement or sufficiency but cannot distinguish graded from thresholded responses. Where quantitative dose–response measurements exist, the titrated variable is a drug or drug combination rather than FOXM1 abundance (16), and therefore do not resolve FOXM1 dosage. A further unresolved issue is isoform composition: the available studies do not independently vary total FOXM1 abundance while controlling the FOXM1b ratio, leaving the molecular definition of FOXM1 “dose” unresolved.

#### 3.1.2 Single-cell resolution

Distinguishing discrete state selection from a graded change in cell-fate probability requires simultaneous measurement of FOXM1 and multiple downstream outputs in individual cells across a range of FOXM1 levels. No such experiment was identified here. Vaz et al. used time-lapse imaging to show that FOXM1 repression increased death in mitosis during antimitotic treatment in MCF-7 cells, whereas the effect was weaker in MDA-MB-231 cells (63). FOXM1 repression did not produce the same death phenotype without antimitotic treatment, indicating dependence on a permissive mitotic-arrest context; in MDA-MB-231 cells, BMF repression was also FOXM1-independent (63). Pan et al. similarly showed in MCF-7 and CAL51 cells that FOXM1 depletion prolonged mitosis and increased mitotic errors, particularly with MAD2 expression, whereas inducible FOXM1 overexpression reduced MAD2-associated lethality, shortened mitotic duration, and altered mitotic-fate distributions in the MAD2-expressing background (64). FOXM1 overexpression alone did not substantially alter mitotic duration or errors, further indicating context dependence (64).

These observations show that FOXM1 modulates single-cell outcomes but do not establish a molecular switch. Mitotic exit versus death are discrete cell-level outcomes regardless of regulatory architecture; a monotonic change in fate probability could therefore mimic a threshold mechanism. Single-cell fate measurements alone consequently do not establish state selection. Senescence provides a related example: Anders et al. showed that CDK4/6 activity stabilizes and activates FOXM1 and contributes to senescence suppression; FOXM1 depletion increased senescence in U2OS cells, whereas constitutively active FOXM1 reduced CDK4/6-inhibitor-induced senescence, with relevance also shown in melanoma and osteosarcoma models (39). These findings support FOXM1-dependent senescence control but not a FOXM1 dosage threshold or biostability.

#### 3.1.3 Time resolution

Testing hysteresis requires FOXM1 to be increased and subsequently decreased through an overlapping range in the same experiment, with transition points measured in both directions. No study identified here has performed such bidirectional FOXM1 titration. Existing evidence consists either of endpoint comparisons between independently derived resistant and parental populations (16, 65), where history is inferred from derivation, or longitudinal measurements during treatment. For example, metabolic imaging of PI3Kα-inhibitor-treated ER-positive breast cancer associated persistent FOXM1 expression with resistance over time (66). Such persistence cannot distinguish hysteresis from a slowly changing graded response. A second, more specific deficit attaches to this axis. Even where FOXM1 has been perturbed over time, the material state of FOXM1 condensates has not been measured at any time point, and no study has reported whether their properties change with age. Because phase-separated assemblies undergo time-dependent changes in viscoelasticity that can render dissolution slow or effectively irreversible (67–70), persistence of a FOXM1-associated output after FOXM1 has fallen is compatible with at least three mechanisms that available designs cannot separate: circuit-level bistability, slow relaxation of a graded response, and kinetic arrest of an aged condensate. Discriminating among them requires that hold time at elevated FOXM1 be varied deliberately and that condensate material state be measured alongside output, as set out in Sections 3.4 and 13.

Two constraints shape the hypothesis. First, FOXM1 abundance alone is insufficient to specify fate: in Vaz et al., FOXM1 repression increased mitotic death in the presence, but not absence, of antimitotic stress (63). Second, identical FOXM1 perturbations can yield different outcomes across backgrounds: the effect on mitotic death was strong in MCF-7 but modest in MDA-MB-231, where BMF repression was FOXM1-independent; Pan et al. likewise showed dependence on MAD2-induced chromosomal instability (63, 64). Dosage models should therefore be conditional on cellular and stress context.

#### 3.1.4 Experimental precedents

Naqvi et al. precisely modulated SOX9 abundance across a continuous range, fitted Hill equations to dependent regulatory elements, and used ED50 and Hill exponent to quantify dosage sensitivity and switch-like behavior (71). Most elements were buffered against modest dosage reduction, whereas a smaller subset was more dosage-sensitive (71). This provides a framework for testing FOXM1 dosage without assuming SOX9-like behavior. The dTAG system establishes rapid, target-specific degradation of appropriately tagged proteins and could be adapted to generate multiple FOXM1 states, although it does not establish FOXM1 dosage sensitivity or hysteresis (72). Finally, single-cell analysis of the upstream Rb–E2F module showed conversion of graded serum input into all-or-none E2F responses and persistence after stimulus removal, consistent with bistability and history dependence (73). This demonstrates switch-like behavior in the broader network, not in FOXM1.

Taken together, evidence supports FOXM1 as a context-dependent regulator of transcriptional, proliferative, resistance, and cell-fate phenotypes, but does not distinguish a graded rheostat, thresholded state selector, or hysteretic switch. “Rheostat,” “state selector,” “threshold,” and “shared attractor” should therefore remain explicit, testable hypotheses. Discrimination requires quantitative FOXM1 titration, simultaneous single-cell measurement of FOXM1 and multiple outputs, and bidirectional perturbation. **Box 1** summarizes the corresponding predictions and discriminating measurements.

Box 1 **Discriminating the rheostat-and-state-selector model from the monotonic-hub null: predictions, required measurements, and current evidence status.**

**Table:** 

Predicted property	Prediction under the rheostat-and-state-selector model	Prediction under the monotonic-hub null	Discriminating measurement	Current status in the FOXM1 breast cancer literature
Dose–response shape	Output programs engage above discrete FOXM1 levels; steep, switch-like response (high Hill exponent).	Output rises smoothly and proportionally across the FOXM1 range.	Graded titration of FOXM1 across ≥5 intermediate levels within one cellular background; Hill-equation fit for each output; comparison of half-maximal dosage (ED_50_) and Hill exponent across outputs.	Not tested. All cited perturbations are categorical (knockdown, deletion or overexpression versus control) (61–63, 108, 119–121, 123, 125, 140–142); the only reported dose–response matrices titrate inhibitor concentration, not FOXM1 abundance (16).
Distribution across cells	At intermediate dose, cells partition into discrete output states showing partial mutual exclusivity.	Unimodal distribution; outputs co-vary continuously within cells.	Simultaneous single-cell measurement of mitotic, metabolic, immune-associated and stem-like output modules across a FOXM1 titration series.	Not tested. Single-cell resolution exists for one output at a time (mitotic fate (63, 64); senescence (39) and demonstrates shifts in the proportion of cells adopting a binary fate, which both models predict.
Path dependence	Switch-on and switch-off levels differ (hysteresis); output persists after FOXM1 falls below the activating level.	Path-independent; a given FOXM1 level yields the same output irrespective of history.	Bidirectional titration (ascending, then descending) within one experiment using reversible FOXM1 control, recording the switching level in each direction.	Not tested for FOXM1 in any system. Endpoint comparison of resistant versus parental lines (16, 65, 165) and longitudinal single-variable imaging (66) cannot separate hysteresis from slow relaxation. Bistability with hysteresis is documented for the upstream Rb–E2F module (73).
Convergence of upstream inputs and parallel collapse of downstream programs	Expected.	Equally expected.	— (not discriminating)	Extensively documented (22, 58, 74–76, 79–84, 95–100). Establishes FOXM1 as a hub; contributes no evidence for dose-dependent state selection.

### 3.2 From abundance to activity: components of a quantitative FOXM1 activity score

If dose is the independent variable, it must be measurable. In breast cancer, functional output is not equivalent to abundance: the genome-wide FOXM1 binding program predicts metastatic outcome better than FOXM1 expression or tumor proliferation index (74). Four candidate determinants may explain this difference (**Box 2**). The metric below is a proposal; it has not been implemented, and each component has measurement limitations.

Box 2 **Components of the proposed FOXM1 activity score in breast cancer: biological basis, candidate measurement, current status and principal limitation of each term.**

**Table:** 

Component	Biological basis	Candidate measurement	Current status in breast cancer	Principal limitation
Total FOXM1 protein *(anchor term)*	The quantity all other terms modify	Per-cell protein quantification against an absolute standard (71)	Inferred almost entirely from bulk mRNA or pan-isoform immunoblot	mRNA is a poor proxy where post-translational stabilization is extensive (74–76, 79–84)
Active-isoform fraction	FOXM1a is transcriptionally inactive; FOXM1b and FOXM1c are active but not equivalent; FOXM1d is cytoplasmic and non-transcriptional (30, 32, 35, 36, 38)	Junction-resolved or long-read quantification across exons Va and VIIa	Unresolved and method-dependent. Isoform-specific qPCR indicated FOXM1b predominance in cancers (38); transcript-level RNA-seq indicated FOXM1c predominance (35)	Not quantified in any breast cancer cohort; FOXM1a is not inert but tumor-suppressive in at least one setting (77), so the term is signed, not merely fractional
Nuclear fraction	Localization is separately regulated and pharmacologically modifiable (36, 49, 78)	Per-cell nuclear-to-cytoplasmic ratio by quantitative immunofluorescence	Reported qualitatively; never combined with abundance into a single metric	Pan-isoform antibodies cannot resolve which isoform is localized, so this term and the isoform term are not independently measurable with current reagents
De-repressed (activated) fraction	NRD–TAD autorepression restricts coactivator access; cyclin–CDK phosphorylation relieves it and progressively raises activity through G2/M (39, 46–48)	Phosphosite occupancy by targeted mass spectrometry, or validated phospho-specific antibodies	Mechanism established; no site validated as a quantitative activity proxy	Activation is graded and conformational; occupancy at any single site may not track transcriptional output
Protein half-life *(determinant, not an independent multiplier)*	Deubiquitinase/E3 balance, ABL1 phosphorylation, O-GlcNAcylation (74–76, 79–84)	Cycloheximide chase or pulse-labelling turnover measurement	Extensively documented (Tier III, Section 6)	At constant synthesis, half-life sets steady-state abundance; entering it beside measured abundance double-counts. It belongs to the abundance term and to the dynamic model, where it governs traversal of a putative threshold
Output anchor *(validation, not a component)*	Functional program, against which any score must be calibrated	FOXM1 cistrome by chromatin occupancy, or a FOXM1 target-gene signature (23, 26)	The cistrome already outperforms FOXM1 expression and proliferation index for metastatic outcome in breast cancer (23)	Bulk-derived; a per-cell equivalent has not been established
Condensate partitioning *(candidate term)*	FOXM1 undergoes liquid–liquid phase separation; condensate disruption lowers oncogenic transcription (6)	Per-cell saturation concentration by quantitative imaging across a concentration series, together with condensate number and volume fraction	Single study, not independently replicated	A further limitation is that this term is treated as a static quantity. Because condensate material properties evolve with time (69, 70), condensate number and volume fraction measured at a single time point do not specify the state of the condensed phase, and measurements should be age-resolved. Fluorescence recovery after photobleaching is the most accessible readout of material state but is not sufficient alone: measured mobility depends strongly on the fitting model adopted and on whether a subregion or the whole condensate is bleached, so the model should be stated explicitly and corroborated by an orthogonal measure where feasible (200).

#### 3.2.1 The active-isoform fraction

Only some FOXM1 protein is transcriptionally competent. Inclusion of both alternative exons disrupts FOXM1a’s transactivation domain, making it inactive, whereas FOXM1b and FOXM1c are active, as confirmed by CRISPR knockout–reconstitution in HEK293T cells (*non-breast, non-cancer*) (35, 75, 76). FOXM1d is predominantly cytoplasmic and signals non-transcriptionally through ROCK, contributing nothing to nuclear transcription (*non-breast: colorectal cancer*) (36). FOXM1b and FOXM1c are also non-equivalent: FOXM1b had greater transforming ability, whereas only FOXM1c was RAF/MEK/MAPK-sensitive through an ERK1/2 target sequence in exon Va (38). FOXM1a is not merely inert: enforced expression suppressed proliferation, colony formation and xenograft growth in OSCC, consistent with tumor-suppressor activity (*non-breast*) (77). Isoform hierarchy remains unsettled: isoform-specific qPCR suggested FOXM1b predominance in cancers (38), whereas GTEx/TCGA transcript quantification placed FOXM1c highest (35). Because isoforms differ by 45- and 114-nt cassette exons, primer placement and short-read deconvolution may underlie this discrepancy; junction-resolved or long-read analysis in breast tumors is needed. No breast-cancer cohort has yet quantified this term.

#### 3.2.2 The nuclear fraction

Competent FOXM1 contributes only after reaching chromatin, and localization is independently regulated. FOXM1d is cytoplasmic (36); c-Src phosphorylation enhances nuclear localization and target-gene expression in luminal B-like breast-cancer models (49); and a small molecule can relocalize nuclear FOXM1 to cytoplasm for autophagic degradation (78). Quantitative immunofluorescence can measure the per-cell nuclear: cytoplasmic ratio, but pan-isoform antibodies prevent independent measurement of isoform and localization in the same cell.

#### 3.2.3 The de-repressed fraction

Nuclear competent FOXM1 can remain inactive because N-terminal negative-regulatory-domain interaction with the transactivation domain restricts coactivator access (47, 48). Cyclin–CDK phosphorylation relieves this autorepression, enables CBP/p300 recruitment and progressively increases activity through G2/M (39, 46–48). Activation is therefore graded. Appropriate readouts are phosphosite occupancy by targeted mass spectrometry or validated phospho-specific antibodies, although no single phosphosite is established as a quantitative proxy for FOXM1 activity in breast cancer.

#### 3.2.4 Protein stability, and why it is not a fourth multiplier

FOXM1 stabilization in breast cancer involves deubiquitinases/E3 ligase (74, 79–83), ABL1 phosphorylation that impedes APC/C-associated ubiquitination (75), and nutrient-sensitive O-GlcNAcylation (76, 84). Stability should not independently multiply a static score because, at fixed synthesis, half-life determines abundance; including both double-counts the same quantity. Instead, stability determines the abundance term (making Section 6 Tier III mechanisms inputs to dose) and is a dynamic rate parameter governing threshold traversal and the ascending/descending-arm hysteresis prediction in Section 3.1.

#### 3.2.5 Non-independence, and formal caution

Terms are not orthogonal. ABL1 phosphorylation affects stability (75); c-Src phosphorylation affects localization and target-gene activity (49); CDK4/6 phosphorylation stabilizes and activates FOXM1 (39); and MEK1 activation of FOXM1c accompanies proteolytic removal of the N-terminal inhibitory domain (38). Thus, a composite score is an empirically calibrated scaffold, not a first-principles formula: a starting form is measured abundance multiplied by active-isoform, nuclear and de-repressed fractions, with fitted exponents and validation against an independent FOXM1 cistrome/target-gene anchor, whose superiority to expression has breast-cancer support (23, 26). Condensate partitioning stands apart from the four terms above and is treated separately in Section 3.3, because phase behavior bears on the form of the dose–response relationship and not only on its input. Disrupting FOXM1 condensates reduces oncogenic transcription (6), but this single, unreplicated study supports condensates as a candidate mechanism rather than as an established component of the score.

Two implementation tiers follow. A research-grade tier for Section 13 titration experiments would combine per-cell protein quantification against an absolute standard (71), isoform-resolved transcripts, nuclear:cytoplasmic ratio and phosphosite occupancy in one system. A tissue-applicable tier for archival cohorts would use isoform-resolved bulk RNA, nuclear-restricted immunohistochemistry and a FOXM1 target-gene output signature. Both remain to be constructed. Finally, the proposal is falsifiable. If this composite does not predict downstream FOXM1-associated output better than total FOXM1 abundance, the compositional claim is wrong and “dose” reduces to abundance; Section 3.1 must then use abundance as its x-axis, with rheostat versus monotonic-hub models adjudicated on abundance alone.

### 3.3 A candidate physical basis for thresholding: phase behavior and the saturation concentration

Section 3.1 predicts that output programs engage above discrete FOXM1 levels and that switching thresholds may differ between increasing and decreasing FOXM1 abundance, but it does not specify a physical mechanism that could generate such nonlinearity. Phase behavior provides a candidate mechanism because phase transitions can create concentration boundaries, while nucleation kinetics can produce metastability and history dependence. FOXM1 undergoes liquid–liquid phase separation, and disruption of its condensates reduces oncogenic transcription (6); the implications of this observation for the dosage model have not previously been developed.

The first constraint is the distinction between total and dilute-phase concentration. In simple phase-separating systems, once the saturation concentration is exceeded, additional protein can be partitioned into the condensed phase while the dilute-phase concentration changes little, providing a buffering mechanism demonstrated experimentally with engineered phase-separating proteins in mammalian cells (85). If transcriptionally relevant FOXM1 activity depended only on free nucleoplasmic concentration and FOXM1 behaved as a simple one-component system with a fixed saturation concentration, further increases in total FOXM1 above that boundary would therefore have limited effects on the free pool. Phase separation would act primarily as a buffer rather than as a continuous rheostat.

Three considerations nevertheless leave a dosage mechanism possible. First, buffering changes the distribution of protein between phases rather than eliminating the dependence of condensate abundance on total protein: above the phase boundary, additional material can increase condensate number or size (85). If FOXM1 output depends on condensate abundance or on the engagement of transcriptional regulatory sites rather than solely on free FOXM1, a dosage-sensitive variable could therefore persist in the condensed phase. This predicts that, above the phase boundary, target-gene output should correlate more closely with condensate abundance or chromatin-associated FOXM1 than with free nucleoplasmic concentration. Second, intracellular condensates are multicomponent systems in which heterotypic interactions can make phase behavior composition-dependent rather than governed by a single fixed saturation concentration (86). FOXM1 also interacts with transcriptional coactivators such as CBP and p300 (46, 48), providing a plausible molecular basis for composition-dependent regulation, although whether these interactions determine the thermodynamics of FOXM1 condensates has not been established. Third, the FOXM1 study links phase separation to FKH consensus DNA elements, indicating that DNA-associated interactions can contribute to condensate formation and transcriptional compartmentalization (6). Thus, the relevant concentration for FOXM1 condensation may not be adequately represented by a single nucleoplasmic average.

These considerations suggest a reformulation of the post-translational layer in which modification can, in principle, alter the phase boundary as well as FOXM1 abundance or activity. In a mean-field description, a modification that changes effective interaction strengths would alter the parameters governing phase separation and hence the concentration at which condensation occurs; for multivalent proteins, however, such behavior is more appropriately viewed through composition-dependent and stickers-and-spacers frameworks than through a single Flory–Huggins parameter (86, 87). Consistent with this possibility, AMPK-mediated phosphorylation within the FOXM1 intrinsically disordered region perturbs condensate formation and reduces oncogenic transcription (6). More broadly, kinase activity can regulate phase transitions of membraneless organelles in cells (88). On this basis, the relevant variable could be conceptualized not as the absolute concentration of active FOXM1, but as its position relative to a context-dependent phase boundary determined jointly by FOXM1 abundance, molecular composition and post-translational state. This provides a physical hypothesis for threshold-like behavior, but it does not establish that FOXM1 transcriptional output is controlled by such a phase boundary. Likewise, nucleation barriers provide a possible source of kinetic history dependence, but whether they generate measurable hysteresis in FOXM1 activity remains untested.

The evidential position is therefore important. A defined saturation concentration or phase boundary for endogenous FOXM1 in breast cancer cells has not been established. The available FOXM1 study demonstrates LLPS, DNA-associated condensation and modulation of condensate formation by AMPK-mediated phosphorylation, but does not establish a quantitative FOXM1 saturation concentration or hysteretic switching behavior (6). The O-GlcNAc-dependent regulation described in Section 6 affects FOXM1 abundance and activity (76, 84), but its effect on FOXM1 phase behavior has not been tested. Nor has it been established whether the phase behavior observed experimentally under the reported conditions quantitatively explains FOXM1 dosage responses at endogenous expression levels in breast cancer cells. Accordingly, the phase-boundary formulation is advanced here as a testable physical hypothesis for the thresholding proposed in Section 3.1, rather than as an established property of FOXM1; the measurements required to distinguish these possibilities are outlined in Section 13. A further limitation of this formulation should be stated before proceeding. The account given above is thermodynamic, and therefore addresses the first prediction of Section 3.1, threshold nonlinearity, but not the third, path dependence: an equilibrium phase boundary yields the same phase whenever the same concentration, composition and modification state recur. The material properties of the condensed phase, rather than the position of the boundary, supply the more plausible candidate substrate for history dependence, and are considered next.

### 3.4 FOXM1 condensate state as a candidate substrate for history-dependent state selection

Recent work places biomolecular condensation within FOXM1 regulation. In breast tumor cells, FOXM1 undergoes liquid–liquid phase separation (LLPS) with forkhead-consensus DNA, compartmentalizes transcriptional machinery, and sustains chromatin accessibility and super-enhancer landscapes required for metastatic outgrowth; AMPK-dependent phosphorylation within an intrinsically disordered region disrupts these condensates and reduces oncogenic transcription (6). In hepatocellular carcinoma, FOXM1 forms dynamic, reversible condensates with ASPM that increase stability and transcriptional activity (89). These studies establish transcriptionally consequential FOXM1 condensation, but they do not determine whether condensate state depends only on the instantaneous FOXM1 level or also on prior exposure.

This distinction is central to a dosage-rheostat/state-selector model. At thermodynamic equilibrium, a coexistence boundary is path-independent; therefore, reproducible separation of assembly and disassembly trajectories would imply multistability or a slowly evolving non-equilibrium variable. One candidate is kinetic asymmetry. In living cells, compatible IDR-containing seeds markedly enhance condensate nucleation and influence where condensates form (90), while phase-separating RNA-binding proteins can form heterogeneous clusters below macroscopic saturation (91). By analogy, prior FOXM1 condensation could lower an apparent re-condensation threshold, accelerate reassembly, or bias reassembly toward previously occupied loci. These FOXM1-specific predictions remain untested. Moreover, slowing the FOXM1 titration rate would be expected to reduce a purely kinetic separation, but rate dependence would not uniquely identify condensate nucleation because slow feedback, protein turnover, post-translational modification, or chromatin remodeling could produce similar behavior.

A second candidate memory variable is condensate material ageing. This evidence is system-specific and should not be generalized to FOXM1. Reconstituted protein condensates examined by Jawerth et al. behaved as ageing Maxwell fluids whose viscosity increased strongly with age (69). In hnRNPA1 low-complexity-domain condensates, sequence-dependent ageing was associated with disorder-to-order transitions and formation of non-fibrillar, β-sheet-containing, semi-crystalline Kelvin-Voigt solids (70). FUS condensates likewise undergo time-dependent conversion toward aggregated states, accelerated by disease-associated mutations (68). These observations establish that condensate age can alter material state, but whether FOXM1 condensates undergo analogous rheological ageing, and whether such ageing changes their transcriptional competence, has not been tested. Importantly, persistence of a FOXM1 assembly would not necessarily imply persistent transcription; ageing could stabilize productive compartmentalization or reduce molecular exchange, so material state and transcriptional output require parallel measurement.

Chromatin may further modulate this process. FOXM1 condensation is already linked to chromatin accessibility (6), whereas chromatin can constrain condensate dynamics: optogenetically generated nuclear condensates show anomalously slow coarsening within the chromatin environment (92); theory predicts that cross-linked chromatin suppresses coalescence and ripening and influences condensate number, size and placement (93); and targeted condensate forces reveal substantial local heterogeneity in chromatin viscoelasticity (94). These findings do not establish locus-specific FOXM1 phase boundaries, but they make locus-dependent nucleation, retention or dissolution kinetics plausible.

The critical prediction is therefore not simply that FOXM1 forms condensates, but that identical FOXM1 levels can support different condensate and transcriptional states depending on prior FOXM1 trajectory or dwell time. This should be tested by matched ascending and descending FOXM1 perturbations while measuring FOXM1 abundance, condensate state, material dynamics, chromatin accessibility and target-gene output in the same experimental framework. Hysteresis or hold-time dependence would support a slowly evolving state variable, but would not by itself prove condensate ageing. If a condensate-dependent state persists after the initiating FOXM1 signal declines, it could provide a biophysical contribution to adaptive plasticity and treatment persistence; a causal role in therapy resistance, however, remains hypothetical.

## 4 Input tier I: genomic and transcriptional priming of the FOXM1 set-point

The abundance of FOXM1 in breast cancer is more accurately treated as a tunable quantity than as a binary on/off attribute, and the first layer that constrains this quantity operates upstream of any RNA- or protein-level control. Before transcripts are stabilized or proteins shielded from degradation, the maximal attainable FOXM1 set-point is shaped by sequence variation and by the transcriptional circuitry in which the gene is embedded. How this ceiling is established frames this section. A recurring theme is that FOXM1 sits at the receiving end of an interconnected transcription-factor network rather than at the head of a linear pathway. Network-based analysis of patient expression data identifies FOXM1, HMGA1 and MYBL2 as mutually reinforcing “switch” genes that modulate one another’s expression (95), and E2F1 occupancy at the FOXM1 promoter can be actively promoted by accessory factors such as CDCA5 (96). These findings collectively support the view that FOXM1 output emerges from coordinated transcriptional inputs.

The cis-regulatory architecture reinforces this picture. FOXM1, together with E2F1 and H3K27ac, occupies an enhancer that drives the effector HJURP, and interfering with that enhancer reduces both HJURP transcription and binding of the transcription-factor complex (97). Read alongside CDCA5-promoted E2F1–FOXM1 binding (96), such data are consistent with enhancer activity and transcription-factor cooperation setting the attainable FOXM1 state rather than merely switching on downstream proliferation. Epigenetic and signaling inputs appear to converge on the same transcriptional layer through different entry points. Integrative mapping of basal-like tumors places FOXM1 among the principal transcription factors embedded in methylation–miRNA–TF feed-forward loops (98), while a tumor-suppressor axis links Hippo–YAP and AKT–FOXO3A signaling to FOXM1 and ERα within a hierarchically organized network (99). That ERα and FOXM1 physically cooperate, with overlapping cistromes at complex enhancers, is further supported by orthogonal evidence from outside the breast (non-breast, non-cancer: pancreatic β-cell) (100).

Across these forms of regulation, sequence variation (101), enhancer activity (97), transcription factor cooperation (95, 96), and epigenetic and signaling control (98–100), a common feature emerges: each acts upstream of FOXM1. Whether these inputs operate hierarchically, additively, or context-specifically remains unresolved; the enhancer hierarchy and the relative weighting of network components are unknown, and the functional consequences of FOXM1 sequence variants remain computational predictions awaiting experimental validation (101).

This multiplicity of upstream regulators is more consistent with a distributed dosage-setting network than with a single dominant pathway, one that plausibly defines the baseline state upon which subsequent RNA-level and post-translational mechanisms act. It also raises the possibility, not yet clinically validated, that network-level indicators, such as E2F/MYBL2 co-expression, could mark FOXM1 dependence more informatively than FOXM1 abundance alone (**Figure 2**). The tuning of this transcriptional baseline is the subject of the next section.

## 5 Input tier II: epitranscriptomic and translational tuning of the FOXM1 set-point

If the genomic tier defines the ceiling of FOXM1 expression, the post-transcriptional layer determines where the needle settles beneath it, adjusting the dose without changing the gene copy number or transcription rate. The literature describes not one dominant regulator, but a set of parallel analog throttles, most converging on triple-negative and endocrine-resistant contexts. The clearest evidence comes from miRNA brakes. miR-548c-3p declines progressively in MCF-7 cells, and its loss tracks with the coordinated upregulation of E2F3 and FOXM1, best read as post-transcriptional derepression rather than *de novo* activation, and is consistent with FOXM1 hypomethylation and poorer survival in triple-negative tumors (102). miR-4521 supplies the complementary experiment: its restoration suppresses FOXM1, perturbs the FOXM1-dependent DNA-damage response, and raises ROS to the point of arrest (103). The fact that two independent miRNAs act as brakes argues for redundancy rather than a single rate-limiting repressor. Directionality, however, remains unresolved. FOXM1 knockdown itself reshapes the miRNA landscape, lowering oncomiR miR-186-5p and de-repressing tumor-suppressor miR-200b-5p (104), a feed-forward loop in which a high-FOXM1 state sustains the environment that keeps it high; therefore, whether a miRNA is cause or consequence depends on where the loop is sampled.

The same braking is relieved by competing endogenous RNAs: lnc-RGS5 sponges miR-542-5p to elevate FoxM1 and VEGFA/Neuropilin-1 (105). The transferable sponge logic, not the specific finding, is reinforced by circTP63, which sponges miR-217 to derepress EZH2 in gallbladder cancer (106), (non-breast: gallbladder cancer; cited as analogy, not as direct FOXM1 evidence), a mechanism identical in grammar even where the target is not FOXM1. A distinct post-transcriptional layer operates through m6A methylation and its readers. The cytoplasmic reader YTHDF1 promotes breast cancer progression by facilitating FOXM1 translation in an m6A-dependent manner (107), whereas the nuclear reader YTHDC1 has been linked to FOXM1-dependent glycolysis in triple-negative disease through CENPA (Section 8) (108). Two readers acting on the same transcript in different subcellular compartments imply a division of labor between nuclear and translational control that has not been resolved. Separately, eIF4E selectively translates structured 5′-UTR transcripts including FOXM1 and, via ERα, raises its transcription, sufficient to confer tamoxifen resistance (109). A further, qualitatively distinct throttle operates at the level of splice-isoform choice. FOXM1 is expressed as several alternatively spliced isoforms, of which FOXM1b and FOXM1c are transcriptionally active while FOXM1a, generated by inclusion of an alternatively spliced exon, is transcriptionally inactive; a CRISPR knockout–reconstitution model confirmed this active/inactive division directly, although in an embryonic kidney rather than a breast background (non-breast, non-cancer: HEK293T), and transcript-level quantification of normal tissues, tumors and cancer cell lines placed FOXM1c highest among the three isoforms (35). This last point is not settled: isoform-specific qPCR together with a literature survey led to the opposite conclusion, that FOXM1b predominates in cancers (38). Because the isoforms differ only by short cassette exons, the discrepancy plausibly reflects the quantification method rather than the biology, and it is treated in Section 3.2 as the principal obstacle to specifying the active-isoform term of a FOXM1 activity score. FOXM1a is not merely inert: in oral squamous cell carcinoma (OSCC) (non-breast: OSCC), its enforced expression suppressed proliferation, colony formation, and xenograft tumor growth, consistent with a tumor-suppressor rather than a neutral role (77). Because these functionally opposed isoforms arise from one gene, exon-inclusion choice, not transcription rate alone, helps set how much active FOXM1 a cell produces. This choice is itself regulated: in OSCC, the splicing factor hnRNP C binds an exonic splicing suppressor to repress inclusion of the FOXM1a-defining exon, shifting the balance toward active FOXM1, with hnRNP C co-expressed with FOXM1 across cancers (77). One implication for the present framework is that bulk measurements of total FOXM1 need not track active-FOXM1 output, since samples with equal total abundance may differ in isoform composition, adding a compositional dimension to FOXM1 “dose.” Importantly, however, this splicing control has been demonstrated in OSCC rather than in breast cancer; while FOXM1 is established as an oncogenic driver across all breast cancer subtypes and the factor most associated with poor survival (110), whether isoform-level splicing regulation operates analogously in breast tumors remains untested, and is flagged here as a specific, tractable gap rather than an established breast-cancer mechanism. These heterogeneous inputs sum onto one analog output, yet their relative weight is unquantified and the m6A-reader division of labor unresolved. The tier nonetheless yields candidate liquid-biopsy surrogates (miR-548c-3p, lnc-RGS5) and one actionable strategy, miRNA-mimic restoration (103) (**Figure 2**).

## 6 Input tier III: post-translational stabilization — the ubiquitin, kinase and glycosylation code

Beyond transcriptional and RNA-level control, FOXM1 abundance is set most proximally at the protein level, where its half-life and activity are continuously adjusted after synthesis. This layer is best understood as a balance of opposing post-translational forces rather than a single switch. The densest body of evidence concerns ubiquitination, where several deubiquitinases converge on the same substrate. USP5 (79), USP7 (80), USP39 (81), and OTUD7B (82) each interact with FOXM1 and limit its polyubiquitylation, whereas the proteasome-associated PSMD14 reduces K63-linked ubiquitination (83); in each case, enzyme depletion accelerates FOXM1 degradation and curtails proliferation, stemness or tumor growth, effects reversed by restoring FOXM1 (80, 82). Conversely, the E3 ligase STUB1 directs FOXM1 toward degradation and thereby relieves FOXM1-dependent transcription (74). Collectively, these observations are compatible with a dynamic equilibrium between stabilizing and destabilizing activities. Notably, the convergence of multiple deubiquitinases on one factor is consistent with redundancy or context-specificity rather than a single rate-limiting enzyme; the current data neither rank these activities nor establish a dominant regulator.

*USP39 requires separate treatment from catalytically active DUBs.* USP39 contains a USP-like domain but lacks the conserved catalytic residues required for canonical USP activity, and biochemical assays have failed to detect significant ubiquitin-cleaving activity; its yeast orthologue Sad1 is likewise catalytically inactive (111–113). Its established function is instead spliceosomal: the human 65-kDa USP39 orthologue is required for recruitment of the U4/U6.U5 tri-snRNP to the pre-spliceosome and formation of mature spliceosomes (114), while USP39 depletion impairs splicing of Aurora B and broader transcript sets, including HMGA2 in ovarian cancer (113, 115). Importantly, Zhang et al. nevertheless showed that USP39 binds FOXM1, prolongs its protein stability and reduces APC/Cdh1-dependent FOXM1 ubiquitination; their own enzymatic assay detected no significant USP39 DUB activity, and their data instead supported competitive interference with APC/Cdh1 binding (81). Thus, the reduction in FOXM1 ubiquitination is experimentally supported, whereas direct catalytic deubiquitination of FOXM1 is not established by these data. This distinction is consistent with the pseudo-DUB concept, in which catalytically inactive DUB-family proteins can exert non-catalytic functions within protein complexes (116). Given USP39’s established role in RNA splicing, an additional effect on FOXM1 RNA processing is plausible but remains untested and should be distinguished experimentally from the protein-stabilizing effect already demonstrated in (81). Accordingly, we retain USP39 as a FOXM1 regulator but annotate it as a catalytically inactive/pseudo-DUB and exclude it from the count of established catalytically active FOXM1 deubiquitinases.

Phosphorylation appears to feed into the same balance. Tyrosine kinase ABL1 phosphorylates FOXM1 at a residue that impedes its recognition by an APC/C-associated ubiquitination step, such that loss of this modification shortens FOXM1 half-life (75). c-Src phosphorylates FOXM1 on tyrosine residues, but the reported consequence is enhanced nuclear localization and target-gene activity, including a feedback loop (49). Taken together, kinase input emerges as a complementary mechanism that can act on either stability or activity, and in the case of ABL1 intersects the ubiquitin layer directly. A third, nutrient-sensitive input is described through O-GlcNAcylation. Reducing O-GlcNAc lowers FOXM1, in one account by elevating SIRT1 to promote FOXM1 degradation (84), and in another by decreasing FOXM1 expression and activity downstream of OGT (76). These convergent findings link the metabolic state to FOXM1 abundance (**Figure 2** and **Table 1**). A related regulatory logic has recently been demonstrated outside breast cancer. In endometrial cancer, oleic acid was shown to interact with HOXB9, reduce its association with the E3 ligase Praja2, and prolong HOXB9 protein stability without increasing its mRNA, thereby promoting ODC1 stabilization and polyamine accumulation (117). This study is cited as a mechanistic analogue, not as evidence for FOXM1. In breast cancer, O-GlcNAc/OGT signaling regulates FOXM1 abundance through mechanisms involving SIRT1 and proteasomal control (76, 84), but direct metabolite–FOXM1 binding and the corresponding half-life mechanism have not been demonstrated. Whether FOXM1 is regulated through a similarly direct nutrient-dependent stabilization mechanism therefore remains unresolved.

Therefore, the available evidence is consistent with a model in which ubiquitin, phosphorylation, and glycosylation inputs operate simultaneously to tune FOXM1, positioning it as a possible integrator of signaling and metabolic states. This interpretation remains provisional: the stoichiometry among competing deubiquitinases is unknown, their hierarchy and whether they act redundantly or context-specifically is unresolved, cross-talk among the three layers has not been systematically examined, and the quantitative contribution of each to FOXM1 abundance and activity remains undetermined. A point of interpretation follows for the dosage framework. Because half-life determines steady-state abundance at a given synthesis rate, the mechanisms described in this tier are determinants of the abundance term of functional FOXM1 dose rather than an additional, independent contributor to it; they should not be entered separately alongside a measured protein level, as set out in Section 3.2. Their distinct contribution is twofold. Kinetically, by setting how rapidly FOXM1 can rise or fall, they govern how quickly the system can traverse a putative threshold, and therefore bear on the hysteresis prediction described in Section 3.1. Thermodynamically, modification of the intrinsically disordered region may additionally move the threshold itself: post-translational modification alters the interactions that drive condensation and thereby shifts the saturation concentration at which FOXM1 demixes, a mechanism established for kinase control of membraneless organelles in other systems (88) and demonstrated for FOXM1 in one report, in which AMPK-mediated phosphorylation of the disordered region perturbed condensate formation (6). On that reading, the modifications catalogued in this tier are not only determinants of how much FOXM1 is present but potential determinants of the concentration at which its behavior changes qualitatively, as developed in Section 3.3. This proposal is at present restricted to phosphorylation; whether O-GlcNAcylation or ubiquitin-linked modification of FOXM1 affects its phase behavior has not been tested.

## 7 Candidate output state I: the mitotic engine and chromosomal instability

The supporting studies derive from molecularly distinct backgrounds whose upstream inputs to FOXM1 differ, and these outputs should therefore not be read as a single program operating uniformly across breast cancer. The distribution of that evidence is itself uneven, and **Table 2** sets it out by subtype, together with the regulatory mechanism, downstream pathway, resistance phenotype, experimental model and evidence grade for each entry. Two features of that distribution qualify the argument developed here. First, the great majority of mechanistic entries derive from basal-like/TNBC or from ER+ models; we identified no study in this literature in which FOXM1 was perturbed in a HER2-amplified breast cancer model, and the one report invoking HER2-dependent FOXM1 expression concerns circulating tumor cells from ER+/HER2-*negative* disease (118), which is a claim about HER2 signaling in non-amplified tumors rather than about HER2-amplified disease. The receptor-tyrosine-kinase leg of a three-subtype scheme is therefore not currently populated, and is flagged in **Table 2** as a gap rather than asserted. Second, where the same perturbation has been applied across subtypes, the result has not always been the same: FOXM1 repression shifted single-cell mitotic fate strongly in MCF-7 but only modestly in MDA-MB-231 cells, in which the relevant effector remained repressed independently of FOXM1 (63). Subtype-divergent inputs converging on a shared output is the observation that motivates this review; it should not be assumed in advance of the evidence for each subtype.

Several distinct effectors illustrate how this output is assembled. FOXM1 has been shown to bind and transcriptionally regulate eukaryotic elongation factor 2 kinase, with its depletion suppressing proliferation, colony formation, migration, and invasion while inducing apoptosis (119). In a separate signaling context, FOXM1 is positioned within an AKT/FOXM1/STMN1 axis upstream of the cytoskeletal regulator STMN1, where its re-expression restores growth otherwise lost upon upstream knockdown (120). FOXM1 also binds the promoter of kinesin KIF23 to promote progression through Wnt/β-catenin signaling (121). The available evidence is consistent with these effectors contributing to a shared proliferative phenotype through partly distinct routes, direct transcriptional control in two instances, and participation in a signaling cascade in another, rather than through a single linear pathway; the data do not establish a hierarchy among them.

At a broader level, a FOXM1-associated set of mitotic and centromeric regulators has been catalogued as overexpressed in androgen-receptor-low and TP53-mutated tumors, correlating positively with proliferation markers and shorter relapse-free survival (122). Notably, the same signature features prominently in chromosomal-instability gene sets, such that high proliferation and chromosomal instability appear together in these tumors (122). These findings are consistent with an association rather than a demonstrated causal relationship, and the current evidence does not establish that FOXM1 directly drives chromosomal instability. A functional study in breast cancer lines qualifies this association in an instructive direction. In human breast cell lines carrying an inducible MAD2 transgene, FOXM1 depletion promoted MAD2-mediated mitotic arrest and exacerbated chromosomal instability, whereas inducible expression of FOXM1b or FOXM1c shortened mitotic duration, reduced mitotic errors and lethality, and restored proliferative fitness; mechanistically, this was attributed to FOXM1-dependent facilitation of mitotic exit through effects on the spindle assembly checkpoint and cyclin B. FOXM1 was itself upregulated after induction of aneuploidy in cells with a dysfunctional checkpoint, and such cells were correspondingly sensitive to FOXM1 knockdown (64). On this evidence, elevated FOXM1 is better read as supporting tolerance of, and fitness recovery from, chromosomal instability than as generating it. That claim is not interchangeable with the correlative co-occurrence of a FOXM1-associated mitotic signature and chromosomal-instability gene sets in androgen-receptor-low, TP53-mutated tumors (122): the former derives from expression-database and survival analyses of tumor tissue and places FOXM1 alongside instability, whereas the latter derives from direct genetic manipulation and indicates that lowering FOXM1 can increase, rather than decrease, mitotic error in a checkpoint-stressed background. This study is also one of the few in this literature to score mitotic outcome at single-cell resolution by time-lapse imaging, and is discussed in that context in Section 3.1.

Taken together, mitotic effectors and instability-associated processes co-occur in these tumors, and their joint dependence on FOXM1 is proposed here, but not demonstrated, to reflect a single selectable output state. The direction of the FOXM1–instability relationship is itself context-dependent, being positive at the level of tumor-tissue correlation (122) and inverse under experimental checkpoint stress (64). The causal relationship between chromosomal instability and later adaptive states is unresolved, and whether such instability drives therapeutic adaptation has not been established. The relative contribution of individual effectors to the overall state is unknown, the hierarchy among downstream modules is undefined, and the extent to which these outputs are co-regulated within individual tumor cells remains uncertain. How this proliferative, genomically diverse state intersects with further output states attributed to FOXM1 is discussed in the following sections.

## 8 Candidate output state II: metabolic reprogramming

Beyond proliferation, the available evidence links FOXM1-associated activity to a program of metabolic rewiring spanning glycolysis, amino acid/glutamine handling, and cholesterol biosynthesis, such that several metabolic processes converge around FOXM1-associated outputs rather than tracking a single pathway (108, 119, 123–127). These arms have each been characterized in particular breast cancer model systems rather than demonstrated uniformly across subtypes; therefore, the metabolic “state” described here is assembled from subtype-restricted observations, and its generality across ER+, HER2+, and basal-like/TNBC disease remains untested.

The arms are partly distinct yet aligned. A glycolytic arm rests principally on FOXM1’s correlative coupling to the centromere protein CENPA, whose restoration recovers glycolysis after FOXM1 silencing (108). A second report assigns a glycolytic role to FOXM1-driven upregulation of squalene epoxidase (SQLE), with enhanced aerobic glycolysis indexed by acidification, glucose consumption, lactate and ATP (125). We report that observation separately from the CENPA arm, and with an explicit qualification, because the pathway assignment it entails is not supported by the known biochemistry of the enzyme and the readouts used do not by themselves establish a change in glycolytic flux; both points are set out in the paragraph that follows. An amino acid/glutamine arm is represented by FOXM1-driven (with FUS) transcription of the amino acid transporter SLC7A5 (LAT1), whose loss lowers glutamine, α-ketoglutarate, and ATP levels, and whose re-expression offsets FOXM1 depletion (123). Relatedly, SLC7A5 has been placed within an SLC7A5/E2F1/PTBP1/PKM2 axis favoring the glycolytic pyruvate-kinase isoform PKM2, providing evidence of crosstalk between amino acid signaling and glycolysis, although in that account, SLC7A5 is driven by E2F1 rather than FOXM1 (124). A cholesterol arm is indicated by evidence that FOXM1 activity supports cholesterol biosynthesis via induction of the sterol-regulatory transcription factor SREBP1, with pharmacological reduction of FOXM1 activity lowering this output (126). A further proliferative–metabolic link is the FOXM1-dependent transcription of eukaryotic elongation factor 2 kinase (eEF2K), which is associated with proliferation and survival (119).

### 8.1 A mechanistic gap in the reported SQLE–glycolysis link

SQLE is a rate-limiting sterol-synthesis enzyme that converts squalene to 2,3-oxidosqualene (128). Xu et al. showed that SQLE perturbation alters ECAR, glucose uptake, lactate and ATP-related readouts, and that SQLE overexpression partially rescues FOXM1-knockdown phenotypes, providing functional evidence for an SQLE–glycolysis association (125). However, these experiments do not identify the biochemical mechanism linking SQLE activity to glycolytic regulation, and ECAR itself cannot be equated with quantitative glycolytic flux because extracellular acidification includes both glycolytic and respiration-derived contributions (129, 130). Several mechanisms are therefore plausible but unproven, including altered citrate/acetyl-CoA handling, NADPH demand, membrane-cholesterol effects on glucose transport, or co-regulation of glycolytic and lipogenic programs downstream of mTORC1/HIF1/SREBP (128, 131–136). Moreover, non-breast studies show that SQLE can exert cholesterol-independent effects through squalene metabolism and mitochondrial or redox regulation (137, 138), underscoring its context dependence. Accordingly, the most conservative interpretation is that SQLE is a sterol-metabolism effector functionally associated with the FOXM1-dependent glycolytic phenotype, while the intervening mechanism and the direction of the underlying metabolic flux remain unresolved; we therefore place SQLE within the cholesterol/sterol arm of the framework while retaining its reported glycolytic association as mechanistically unresolved (125, 139).

Notably, these outputs are linked to survival under stress: FOXM1-associated glycolytic activity has been connected to suppression of anoikis (125), and shared energy-related readouts are consistent with a contribution to fitness (123, 125), although such relationships remain associative. Orthogonal support comes from a non-breast setting, where FOXM1 correlates with glycolytic and *de novo* lipogenic genes and modulates glucose uptake (127); (non-breast: colorectal cancer); this broadens plausibility across contexts but does not establish universal conservation.

Across these branches, glycolytic, glutamine, and cholesterol outputs may constitute a coordinated FOXM1-associated metabolic state supporting growth and adaptation, although this does not demonstrate that FOXM1 is a metabolic master switch (**Figure 4**). Whether these outputs are co-selected with other FOXM1-associated programs is unresolved; their hierarchy is unknown; the contribution of each branch is unestablished; and whether they coexist within individual cells or are instead subtype-restricted arms observed in different model backgrounds remains uncertain. The intersection of this metabolic state with subsequent outputs is discussed next.

**Figure 4:**
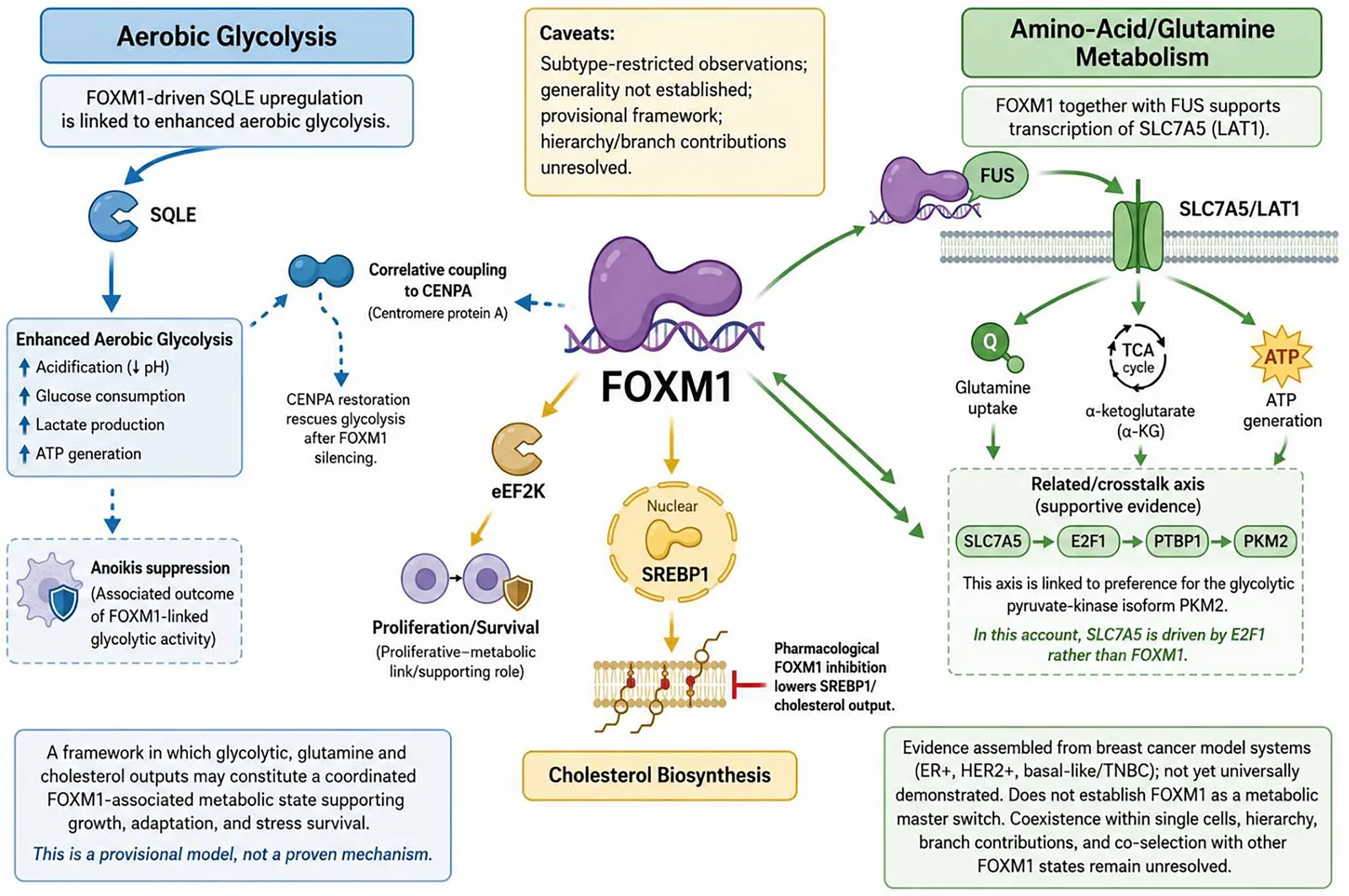
FOXM1-associated metabolic reprogramming in breast cancer. FOXM1-linked activity converges on aerobic glycolysis, amino acid/glutamine handling and cholesterol biosynthesis through CENPA, FUS–SLC7A5/LAT1, SREBP1, SQLE and eEF2K-associated pathways. These outputs are associated with increased glucose consumption, lactate and ATP production, glutamine utilization, α-ketoglutarate generation, proliferation and stress survival, while crosstalk with the SLC7A5/E2F1/PTBP1/PKM2 axis may further support glycolytic metabolism. SQLE is placed within the cholesterol arm, consistent with its established function as the second rate-limiting enzyme of the mevalonate–sterol pathway; the dashed arrow to the glycolytic output denotes a reported association (125) for which no intervening mechanism has been identified and for which the supporting readouts do not distinguish glycolytic from oxidative flux (Section 8). Because the evidence derives from subtype-restricted breast cancer models, this integrated metabolic state remains provisional and does not establish FOXM1 as a universal metabolic master regulator.

## 9 Candidate output state III: immune evasion and microenvironmental reprogramming

The output states considered so far are largely tumor-intrinsic. The available evidence indicates that FOXM1-associated activity also extends outward to shape the immune microenvironment, coupling tumor-cell programs to host immune responses rather than keeping them separate (140–143). Taken together, these observations are compatible with an immune-active output state, although the degree of co-regulation among its components remains undefined. The densest line concerns macrophages, where FOXM1 activity is consistently associated with a pro-tumor, M2-skewed compartment that lowering FOXM1 reverses. Silencing tumor-cell FOXM1 upregulates KLF15, reduces M2 infiltration, and repolarizes macrophages toward an M1 phenotype, reportedly enhancing immune-checkpoint-inhibitor efficacy (140). A second route is intercellular: FOXM1 carried within TNBC-derived exosomes raises FOXM1 in recipient macrophages, where it transcriptionally activates IDO1 and, in this account, suppresses ferroptosis to favor M2 polarization (141). A third links FOXM1 to transcriptional activation of KIF20A, again driving M2 polarization and, through it, invasion and metastasis (142). The common theme is bidirectional tumor–macrophage communication, including via exosomal transfer, although the effectors invoked, a KLF15 axis, an IDO1/ferroptosis axis, a KIF20A axis, differ across reports and have not been reconciled.

A separate line addresses innate immunity. Tumor-intrinsic FOXM1 has been reported to epigenetically silence the DNA-sensing protein STING through a DNMT1–UHRF1 complex, limiting CHOP-dependent induction of the stress ligand ULBP1 and weakening NKG2D-mediated natural-killer and T-cell cytotoxicity, with higher FOXM1/DNMT1 and lower STING/ULBP1 associated with poorer survival, reduced immunotherapy responsiveness, and impaired immune memory (143). Notably, this finding is represented by a full report and a closely matching conference abstract of the same work (143); these constitute one line of evidence, not independent confirmation, and any vaccine-related extension is a proposed implication rather than a separate dataset. A third, mechanistically distinct output concerns checkpoint regulation, but its provenance must be stated precisely. Inhibition of AKT-dependent FOXO3a phosphorylation was reported to lower FOXM1 and suppress PD-L1 transcription while raising interferon-stimulated genes and MHC-I, positioning FOXM1 as supportive of checkpoint-ligand expression; the FOXO3a- and FOXM1-dependence of this effect was, however, established in gastric and colorectal cancer cells, and the study contains no breast cancer model (non-breast: gastric and colorectal cancer) (144). It is cited here for its transferable mechanism and not as breast cancer evidence, and the checkpoint arm should accordingly not be counted among the breast-derived components of this output state.

Thus, a FOXM1-associated state may couple tumor-intrinsic biology to several arms of immune evasion (**Figure 5**). However, the evidence is unevenly distributed across subtypes: the macrophage-education arm is anchored in triple-negative models, including exosomal FOXM1 transfer (140–142), and the innate-sensing (STING) arm is likewise TNBC-derived, having been established by CRISPR/Cas9 FOXM1 knockout in triple-negative cell lines and syngeneic mouse models (143). The checkpoint (PD-L1) arm, by contrast, rests on non-breast models (144) and is retained only as a transferable mechanism. On the present evidence, therefore, the immune-evasion state is supported by two breast-derived arms, both in TNBC, rather than by three arms spanning breast cancer generally; whether either arm operates in ER+ or HER2-amplified disease is untested (**Table 2**).

**Figure 5:**
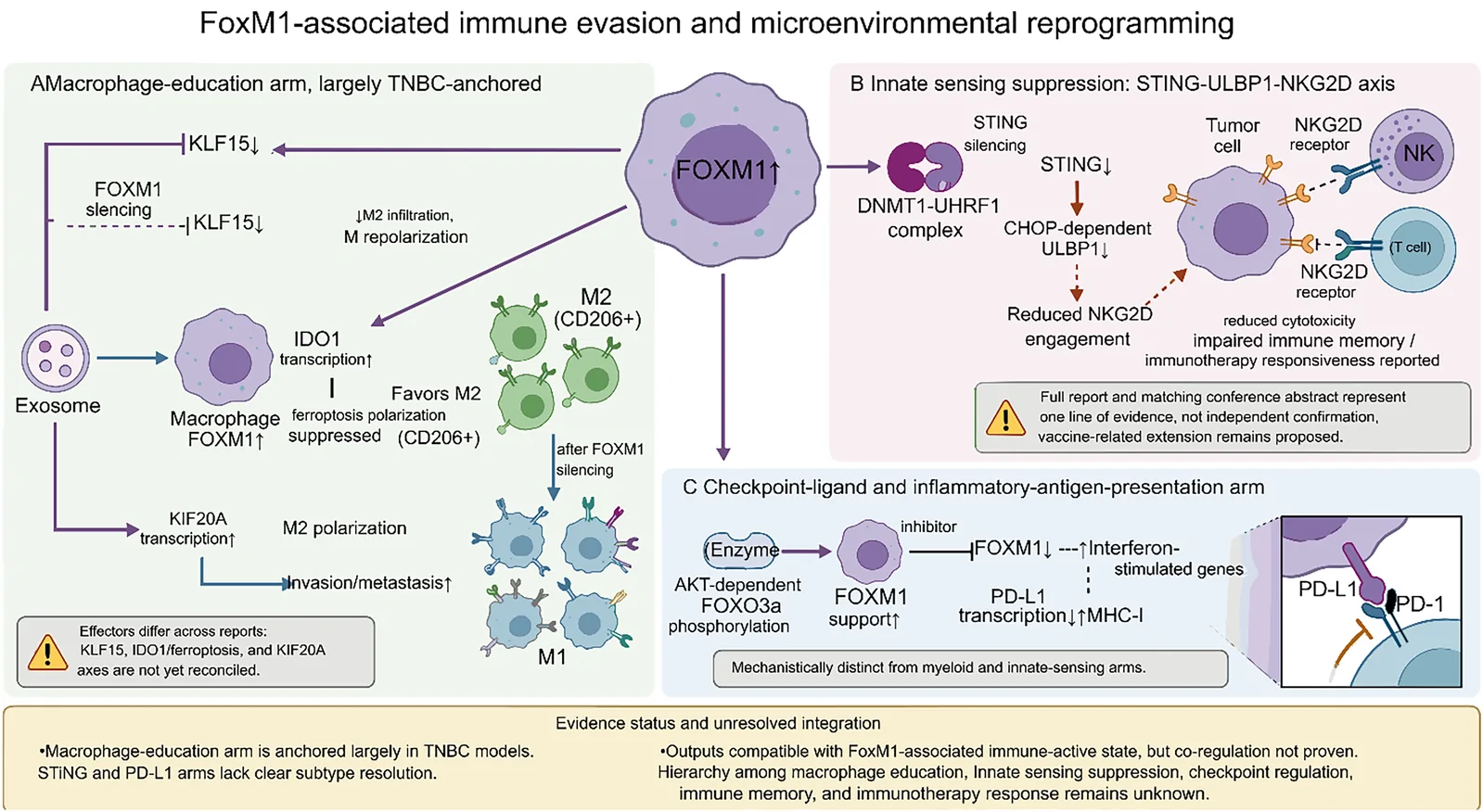
FOXM1-associated immune evasion and microenvironmental reprogramming. FOXM1 is linked to M2-skewed macrophage education, DNMT1–UHRF1-dependent STING–ULBP1–NKG2D suppression, and support of PD-L1 expression. These findings suggest a provisional immune-evasion state, but the three arms are mechanistically distinct, unevenly resolved across breast cancer subtypes, and not yet proven to form one coordinated pathway.

## 10 Candidate output state IV: adaptive and stem-like programs (plasticity, EMT, dedifferentiation)

In addition to its canonical proliferative output, FOXM1 activity has been repeatedly associated with a cellular state defined by phenotypic flexibility rather than a fixed identity. A recurring observation is that elevated FOXM1 levels are accompanied by the suppression of differentiation: disruption of the FoxM1/Rb repressive interaction expands differentiated alveolar-type tumor cells and diminishes the prometastatic compartment, linking FOXM1’s repressive arm to the maintenance of an undifferentiated, metastasis-competent phenotype (145). Converging with this, multiple signaling contexts position FOXM1 downstream of or coupled to stemness programs. FOXM1 has been associated with stem-like behavior and migration in a YAP1/Hippo-dependent manner, with modulation of OCT4 and NANOG transcription (146), and with GDF-15–dependent stemness, in which FOXM1 expression tracks with OCT4, SOX2, and the drug-efflux marker ABCC5 (147). In triple-negative models, FOXM1 appears within a CSC-associated marker set (alongside SOX2, SOX9, and NANOG) regulated through a CUEDC2/β-catenin/OCT4 axis (148), while reduced CLDN11 elevates FOXM1 via Hedgehog signaling to sustain proliferative and migratory traits (149).

These observations span distinct upstream inputs, Hedgehog (149), YAP1 (146), GDF-15 (147), β-catenin/OCT4 (148), yet converge on overlapping outputs: stemness markers, migration, and impaired differentiation. One feature of these inputs warrants separate consideration because it introduces a regulatory dimension not captured by the present framework. YAP1 has been implicated in breast cancer stemness and in the regulation of FOXM1 within the RASSF1A–YAP1–AKT1–FOXO3A network (99, 146). More broadly, YAP and TAZ are major nuclear mediators of mechanotransduction, translating extracellular-matrix rigidity, cell shape and cytoskeletal tension into transcriptional responses (150). Thus, if mechanical cues regulate FOXM1 through YAP1, the FOXM1 set-point may be influenced not only by biochemical signaling but also by the physical state of the cellular microenvironment. This possibility is currently not incorporated into the dosage framework. It should therefore be regarded as a candidate, unmeasured input rather than as an established determinant of FOXM1 dose, because the direction and magnitude of any direct mechanosensitive control of FOXM1 in breast cancer remain unresolved. The available evidence is consistent with convergence rather than a defined hierarchy, and the relationships among these contexts remain unresolved. Notably, the partial (hybrid) EMT state offers a partial mechanistic frame, where transient Wnt/ERK/CDK4/6 activation couples FOXM1-associated self-renewal to retention of a hybrid identity and active suppression of differentiation (151). Taken together, these findings are compatible with a plastic, potentially reversible adaptive state in which proliferation, migration, de-differentiation, and self-renewal are co-regulated. Within the limits of current data, whether these represent a single coordinated program or partially independent modules is undetermined, and reversibility has not been directly examined. The link between this plastic state and resistance remains largely inferential. One study reported FOXM1-associated stemness alongside *intrinsic* drug resistance through ABCC5 (147); however, this should be distinguished from acquired resistance, which these data do not address, a connection developed in the following section.

## 11 Convergence: FOXM1 as a candidate shared attractor of therapy resistance

Before turning to specific therapeutic contexts, the sense in which FOXM1 is described here as a “shared attractor” warrants precise definition, as the term is intended in its dynamical-systems meaning rather than as a loose metaphor. In the gene-regulatory-network formalization of Waddington’s landscape, a cell state corresponds to an attractor, a stable configuration toward which the system settles from a range of initial conditions, and its basin of attraction is the set of starting states that flow into it. To call FOXM1-associated output a shared attractor of resistance is therefore a specific and falsifiable claim: that mechanistically distinct therapies, representing different initial conditions and trajectories, converge on the same stable transcriptional state rather than on separate end-states that bulk averaging merely renders superficially similar. This is a dynamical assertion about convergence and stability, and is thus stronger than describing FOXM1 as a network “hub,” which is a static, topological property.

The formalism is not original to this article; representing cell fates as attractor states of a high-dimensional gene-regulatory network is an established framework (152). What it demands, however, is a level of evidence that must be distinguished from three weaker claims with which it is easily conflated, and the evidence assembled below should be assigned to the appropriate level rather than read as supporting the strongest. The weakest claim is *phenotypic convergence*: mechanistically distinct therapies produce a shared functional outcome, namely survival under treatment. This is documented throughout Section 11.2 and is uninformative about molecular state, since unrelated mechanisms can produce identical survival.

The second is *marker convergence*: a named molecular feature, here FOXM1-associated output, is elevated in each resistant population. This is what **Table 3** records, and it is the level at which almost all of the present evidence sits. Its limitation is that “FOXM1-associated output” is operationalized differently across the cited studies, as FOXM1 transcript, protein, or a downstream effector, so that two populations may both qualify as FOXM1-high while sharing little else. A shared marker is not a shared state.

The third is *transcriptional-state convergence*: independently derived resistant populations occupy overlapping regions of global expression space. Establishing this requires joint profiling of those populations within a single experiment against a shared reference. No cited study does so; each characterized its own resistant line against its own parental control, so the cross-study similarity described here is inferred from separate gene lists rather than measured. This level is additionally vulnerable to a specific artefact of bulk measurement: populations with different mixtures of single-cell states can yield indistinguishable population means, so apparent convergence may be produced by averaging rather than by biology.

Only the fourth level, *dynamical convergence*, corresponds to the attractor claim, and it has three requirements. Trajectories originating from distinct initial conditions must be shown to approach a common region of state space, which requires measurement over time rather than at endpoint. The resulting state must be stable, meaning maintained after the forcing that produced it is withdrawn; a transcriptional configuration held in place by continuous drug exposure is a forced state, not an attractor of the untreated system. And the basin must admit a range of starting conditions. Every resistant model cited in this section was derived and maintained under continuous drug pressure, and none was profiled following drug withdrawal, so the stability requirement is entirely untested. That this matter is not speculative: in a lineage-barcoded persister system, both cycling and non-cycling drug-tolerant cells rapidly reacquired drug sensitivity once the drug was removed (*non-breast: lung and other carcinoma models*) (153). Accordingly, the evidence below establishes convergence at the second level, is suggestive at the third, and does not address the fourth; the attractor language should be read as a structured, testable hypothesis rather than a demonstrated property (**Table 3**).

### 11.1 Endocrine/ER+ signaling resistance

A recurring theme across mechanistically distinct therapeutic pressures in ER+ breast cancer is that resistance coincides with the persistence or reactivation of FOXM1-associated transcriptional output rather than its loss. In the endocrine setting, FOXM1 acts as a direct transcriptional target of ERα, and its silencing abolishes estrogen-driven proliferation and reverses acquired tamoxifen resistance, whereas its enforced expression overrides anti-estrogen–mediated arrest (25). Notably, this same output is sustained through divergent upstream routes: amplification of ERα activity via RGC-32/PI3K (154), retention of ERα signaling by amphiregulin–EGFR–PI3K/Akt/mTOR under aromatase-inhibitor pressure (155), and a switch from ERα-dependent to HER2-dependent, NF-κB-supported FOXM1 expression in circulating tumor cells that have escaped endocrine control (118). These observations are compatible with convergence on a shared output, even where the upstream wiring differs.

A parallel pattern emerges under PI3Kα/AKT inhibition. Resistance to PI3Kα inhibition is associated with persistent FOXM1 expression following treatment, which reprograms downstream metabolism (66), while AKT-inhibitor efficacy tracks with a FOXO3-dependent reduction in FOXM1, such that FOXO3 loss or sustained FOXM1 confers resistance (156). Taken together, the available evidence is consistent with FOXM1 occupying a recurrent node at which distinct therapeutic insults are buffered by maintenance of its program rather than by entirely separate effectors.

Importantly, several of these contexts also report restoration of sensitivity through FOXM1 downregulation (156), NF-κB inhibition (118), or p110α destabilization via the N6AMT1–NEDD4L axis (157), findings compatible with partial reversibility, although whether this reflects a true state transition remains unresolved. Within the limits of the current data, whether endocrine- and PI3K/AKT-associated resistance corresponds to the same molecular state and how these adaptive routes are hierarchically related cannot be determined; single-cell-resolution and kinetic evidence are lacking, and the observed similarities may reflect convergence or only partial overlap.

### 11.2 DNA-damage, PARP, chemotherapy, radiation, CDK4/6 and FOXM1-inhibitor resistance

A consistent observation across mechanistically unrelated therapeutic pressures is that residual or reactivated FOXM1-associated output marks a resistant phenotype, whereas its suppression tends to restore drug responsiveness. This pattern recurs whether the insult is anthracycline chemotherapy, where FOXM1 sustains doxorubicin resistance by maintaining DNA repair gene expression and partnering with NF-κB to limit DNA damage (61); radiotherapy, where an ACSL4–FOXM1 axis supports radioresistance and a stem-like CD24^-^/CD44^+^ phenotype, and FOXM1 inhibition restores radiosensitivity by impairing the DNA-damage response (62); or antimitotic agents, where high FOXM1 represses the pro-apoptotic effector BMF to limit death in mitosis, such that FOXM1 repression shifts individual cells, scored by time-lapse imaging, towards mitotic death; notably, this FOXM1-dependence was evident in MCF-7 cells but largely absent in anoikis-resistant MDA-MB-231 cells, in which BMF remained repressed independently of FOXM1, indicating that the same FOXM1 change does not produce the same output across breast cancer backgrounds (63). Although the downstream effectors differ, these contexts converge on FOXM1 as a node that couple’s survival, repair, and apoptotic thresholds.

The re-sensitization literature reinforces this theme through interventions that share little beyond a common endpoint. Suppression of FOXM1, via flavopiridol acting on FOXM1/NFκB and RAS/MAPK with reduced repair capacity (158) (reported in a preprint that has not completed peer review), morin acting through EGFR/STAT3 with attenuated RAD51 and survivin (159), or maslinic acid acting through a MELK–FoxM1–ABCB1 cascade that lowers drug efflux (160), repeatedly restores chemotherapy sensitivity. A parallel logic operates in the PARP-inhibitor setting, where olaparib provokes adaptive upregulation of FOXM1 alongside repair factors, and where lowering FOXM1 either pharmacologically (FDI-6) or through PI3K inhibition (BKM120, via FOXM1/Exo1) converts BRCA-proficient tumors to an HR-deficient, olaparib-sensitive state (161, 162). Notably, FOXM1 co-occupies HR-gene regulatory regions with MYC, and BET inhibition disrupting this axis induces a BRCAness phenotype (163); however, sustained FOXM1 silencing can trigger compensatory MYC upregulation that preserves repair output, indicating that the network is buffered rather than linear.

This buffering is most evident where FOXM1 itself is the target. Acquired resistance to FOXM1 inhibitors is accompanied by suppression of ferroptosis and apoptosis and elevated stem-cell markers (16), and by transcriptional genome rewiring that reverses the FOXM1 network while activating inflammatory and HER2/EGFR programs (65). Comparable adaptation underlies CDK4/6-inhibitor resistance, where FOXM1 and PLK1 are co-regulated within the RB1 pathway (164) and where FOXM1- and CDK4/6-resistant states share an elevated JAK/STAT1–interferon–ISGylation network, yet retain cross-sensitivity that permits sequential targeting (165). As complementary evidence, the related forkhead factor FOXA1 also modulates doxorubicin and paclitaxel sensitivity (166), consistent with broader forkhead participation in drug-response phenotypes without implying an identical mechanism.

On balance, distinct therapies appear to intersect with overlapping FOXM1-associated adaptive processes. The re-sensitization findings are compatible with partial reversibility, although whether they reflect true state transitions remains unresolved. Two observations in this section constrain the attractor hypothesis and require explicit treatment. First, resistance to FOXM1 inhibition is accompanied by reversal of the FOXM1 transcriptional network, together with rewiring toward inflammatory and HER2/EGFR programs and reduced ERα (65). This indicates that FOXM1-associated output is not the only accessible resistance configuration under therapeutic pressure. A more precise formulation is therefore that FOXM1-associated output represents one possible resistance configuration among several, with the accessible state depending on the node targeted by therapy. This predicts that resistance states should differ systematically according to whether FOXM1 itself or another network node is targeted, a proposition testable by comparing resistant populations generated under the two conditions.

Second, shared regulatory features do not necessarily define a common state. FOXM1-inhibitor- and CDK4/6-inhibitor-resistant cells both show increased JAK/STAT1–interferon–ISGylation signaling, yet retain differential drug sensitivities that permit reciprocal sequential targeting (165). Thus, a shared network module does not establish occupancy of a common attractor. Within the current evidence, whether these resistance phenotypes constitute a single molecular state remains unresolved; genome rewiring demonstrates plasticity but not a common resistant state, while the hierarchy among the adaptive networks remains undefined.

## 12 Drugging the proposed rheostat: therapeutic modalities against FOXM1

One point should be stated plainly before the individual modalities are surveyed, because it conditions how all of them should be read. Despite sustained interest in FOXM1 as a therapeutic target and steady progress in inhibitor development, there are currently no FDA-approved FOXM1 inhibitors, and none has advanced to clinical trials (24). FOXM1-targeting agents developed to date have been characterized in cell-based and mouse models, where some have shown promising efficacy and safety, but their translation to the clinic remains a future goal rather than a current reality (167); the small-molecule inhibitors assembled in this field accordingly span various stages of preclinical development, and their advancement continues to face substantial challenges (168). For a breast cancer readership, the practical implication is that FOXM1 remains an investigational target rather than a clinically actionable one. The modalities that follow, direct inhibitors, degraders, condensate disruptors, indirect pathway suppression, and targeted delivery, should be read as preclinical strategies at differing stages of development, not as options presently available at the bedside (**Figure 6** and **Table 4**). A final point should be stated plainly when interpreting the modalities surveyed above. Reduction of FOXM1 abundance or activity does not, by itself, establish that an agent’s antitumor effect is FOXM1-dependent, particularly when additional targets or pathways are also affected (169). We therefore distinguish FOXM1 modulation from target attribution using four criteria: direct biophysical engagement of FOXM1; a selective molecular consequence consistent with FOXM1 inhibition; genetic epistasis or rescue evidence supporting FOXM1 dependence; and, where available, an *in vivo* pharmacodynamic measure of target engagement. These criteria define four evidence tiers in **Table 4**. Tier 1 requires direct binding, selective molecular effects, and genetic evidence of dependence; Tier 2 includes agents with evidence of FOXM1 targeting but incomplete validation; Tier 3 includes agents for which independently documented activities confound attribution; and Tier 4 includes agents that reduce FOXM1 downstream of an upstream pathway without isolating its specific contribution. The tiers therefore grade the strength of evidence for FOXM1 target attribution, not therapeutic efficacy.

**Figure 6:**
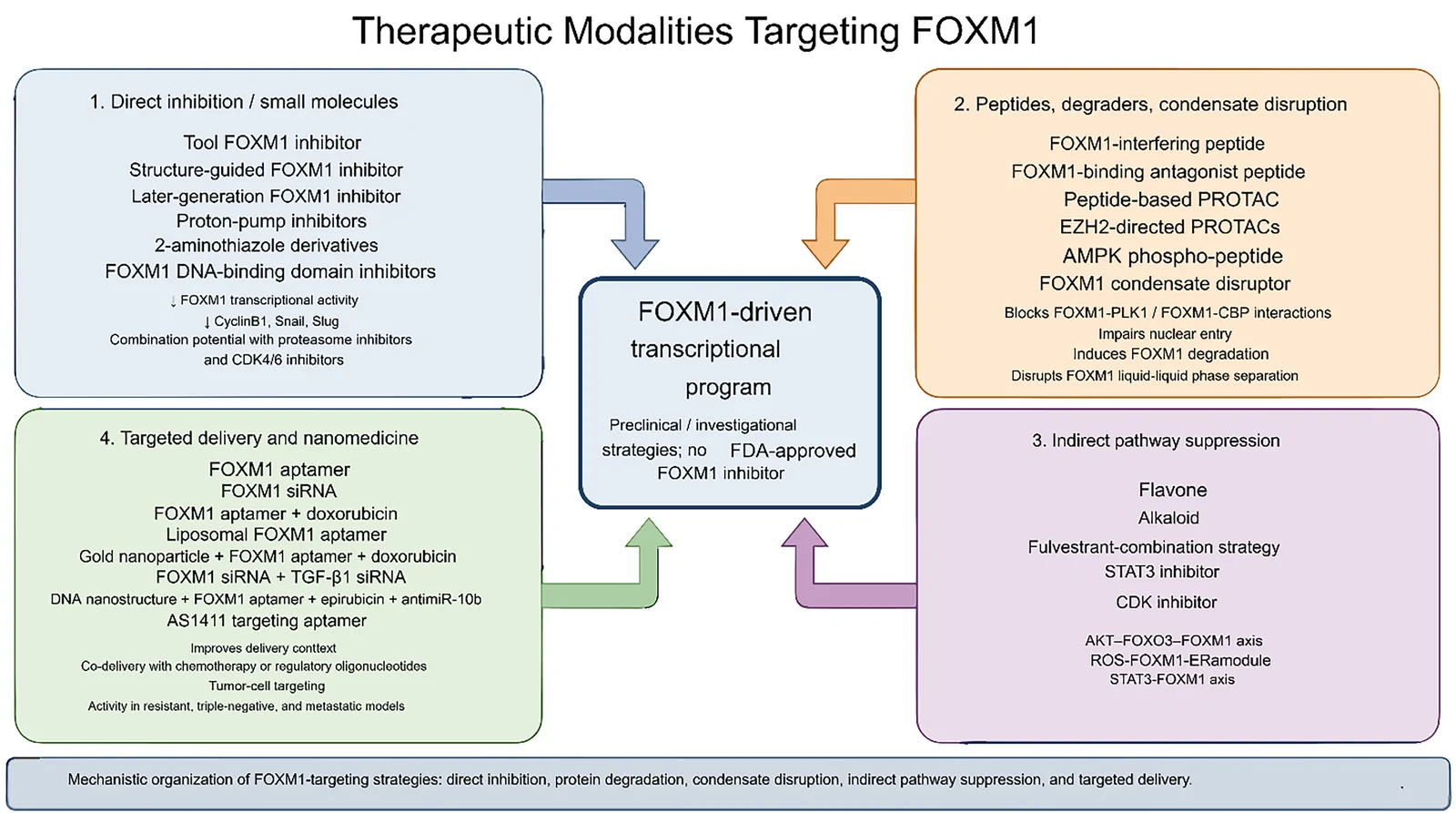
Therapeutic modalities targeting FOXM1. FOXM1-directed strategies include direct small-molecule inhibition, peptide interference, protein degradation, condensate disruption, indirect pathway suppression, and targeted delivery using aptamers, siRNA, and nanomedicine. These approaches reduce FOXM1 activity or abundance through distinct, complementary mechanisms; however, they remain preclinical, with no FDA-approved FOXM1 inhibitor or clinical trial validation to date.

**Table 4:** FOXM1-directed therapeutic modalities in breast cancer, organized by mechanism of engagement. All entries derive from breast cancer.

Agent	Mechanism of engagement	Chemical/platform class	Key effect	Preclinical stage	Evidence tier for FOXM1 attribution	Ref.
Direct small-molecule inhibitors and repurposing						
FDI-6	Direct inhibition	Small molecule (tool inhibitor)	Downregulates FOXM1 and targets CyclinB1, Snail, Slug; suppresses proliferation, migration, and invasion with increased apoptosis; synergizes with doxorubicin.	*In vitro* (2D/3D)	Tier 1. Engagement line present: direct binding to FOXM1, displacement from genomic targets in MCF-7 cells, and RNA-seq selectivity for FOXM1-activated genes with occupancy confirmed by ChIP-PCR, established in the original characterization (170). Epistasis not reported. The validation resides in (170), not in this application study.	(171)
In silico–identified inhibitor	Direct inhibition	Small molecule (structure-guided)	Binds FOXM1 in docking/MD studies; suppresses FOXM1 expression, proliferation, migration and invasion with apoptosis; more potent than FDI-6.	*In vitro* + *in vivo* (xenograft)	Tier 2. Computational docking and molecular dynamics only. No biophysical binding assay, no chromatin-displacement data, no FOXM1-depletion control. Reported in abstract form only and not counted as independent evidence (Section 3).	(172)
STL001	Direct inhibition	Small molecule (next-gen STL427944 analog)	Up to ~50× more efficient than its predecessor; suppresses endogenous and therapy-induced FOXM1; and broadly sensitizes cancers to conventional therapies.	*In vitro*	Tier 1. Epistasis line present: the sensitizing effect is abolished in cells already depleted of FOXM1, excluding an FOXM1-independent route to the same endpoint. Direct biophysical binding assay not reported. This is the only genetic FOXM1-dependence control in the agents surveyed here.	(173)
STL427944	Degradation (autophagic)	Small molecule (network-nominated)	Relocalizes nuclear FOXM1 to the cytoplasm and promotes autophagic degradation; sensitizes cells to platinum agents, 5-fluorouracil, and taxanes.	*In vitro*	Tier 2. Relocalization and autophagic loss of FOXM1 are documented, but the proximate molecular target is not established and no FOXM1-depletion control is reported; the compound was nominated by network analysis rather than designed against FOXM1.	(78)
Pantoprazole/Rabeprazole	Direct inhibition (repurposing)	Small molecule (proton-pump inhibitors)	Bind FOXM1 and inhibit its activity and downstream eEF2K signaling; reduce breast cancer cell proliferation	In silico + *in vitro*	Tier 2. Binding inferred from docking together with a downstream signalling change; no chromatin-displacement or depletion control. These are approved agents whose established primary pharmacology is at the gastric H^+^/K^+^-ATPase, so FOXM1-independent antitumor activity is not excluded.	(174)
Connectivity-Map compounds	Direct inhibition (computational hits)	Small molecules (bioinformatic)	The predicted reverse of a FOXM1-high/PPARA-low signature was found to have 19 candidates nominated but not yet experimentally validated.	In silico	Tier 2. Computational signature-reversal nomination only. No experimental validation of any kind is reported, and engagement of FOXM1 is predicted rather than tested.	(175)
2-Aminothiazole derivatives (C11)	Direct inhibition	Small molecule (*de novo*, FOXM1-DBD)	C11 binds to the FOXM1 DNA-binding domain and suppresses transcriptional activity, outperforming FDI-6 in TNBC cell lines.	In silico + *in vitro*	Tier 2. Rationally designed against the FOXM1 DNA-binding domain with docking support and a transcriptional-activity readout, but neither genome-wide selectivity nor FOXM1-dependence of the phenotype was tested.	(176)
FOXM1 inhibitors + targeted drugs	Direct inhibition (combination)	Small molecule (1,1-diarylethylene)	Synergy with proteasome inhibitors (all subtypes) and CDK4/6 inhibitors (ER+), enhanced G2/M arrest and apoptosis, and enabled dose reduction.	*In vitro*	Tier 2. Synergy with proteasome inhibitors requires careful reading: proteasome inhibitors themselves suppress FOXM1 transcriptional activity and expression (60), so this pairs two agents that each lower FOXM1 rather than demonstrating cooperation with an orthogonal mechanism.	(169)
Peptides, degraders and condensate disruption						
M1-NP1 peptide	Functional interference	Interfering peptide	Binds the FOXM1 C-terminus, disrupts PLK1 and CBP interactions, blocks nuclear entry, and suppresses transcriptional activity; low immunogenicity/hemolysis.	*In vitro* + *in vivo* (WT mice)	Tier 2. Physical engagement is supported by a defined binding region and disrupted protein–protein interactions, but neither genome-wide transcriptional selectivity nor loss of phenotype in FOXM1-depleted cells was tested. Section 12.2 describes M1-21; M1-NP1 is a distinct, related construct.	(194)
FOXM1-PROTAC	Degradation	Peptide-based PROTAC (antagonist peptide + pomalidomide + TAT)	Degrades FOXM1; inhibits viability, migration, and invasion, and suppresses xenografts; lowers GLUT1 and PD-L1 as downstream readouts.	*In vitro* + *in vivo* (xenograft)	Tier 2. Degradation of FOXM1 is demonstrated, but the phenotype was not shown to require FOXM1, and the GLUT1 and PD-L1 changes are downstream observations within the same study rather than independent target validation.	(178)
EZH2 PROTACs (MS177, MS8815)	Degradation (node)	Small-molecule PROTAC	Degrade the EZH2–FOXM1 node, thereby reducing shared target-gene expression; remains active even in tamoxifen-resistant cells.	*In vitro*	Tier 4. The agent is directed against EZH2; the reduction in FOXM1 is a node-level consequence of degrading a different protein. No FOXM1-specific contribution to the phenotype has been isolated.	(179)
AMPK agonist/phospho-peptide	Condensate disruption	Small molecule (AMPK agonist) + peptide	AMPK phosphorylates FOXM1 IDR to perturb liquid–liquid phase-separated condensates, reducing oncogenic transcription, and restoring tumor immunogenicity.	*In vitro* + *in vivo*	Tier 2 (phospho-peptide arm: engages the FOXM1 intrinsically disordered region directly). Tier 3 (AMPK-agonist arm: AMPK activation carries extensive independent activity sufficient to produce the same phenotype). Single study, not independently replicated.	(6)
Indirect and natural-product–mediated suppression						
Apigenin	Indirect	Natural product (flavone)	It inhibits Akt/FOXM1 signaling in an Akt-driven endocrine-resistant context and induces G2/M arrest and apoptosis.	*In vitro*	Tier 4. FOXM1 reduction is a measured consequence of AKT/FOXO3 perturbation. That node independently controls proliferation and survival through effectors other than FOXM1, and no FOXM1-depletion or refractory-rescue control was reported.	(182)
Chelidonine	Indirect	Natural product (alkaloid)	It acts through the AKT/FOXO3/FOXM1 axis, lowering FOXM1 and PLK1 levels, and driving mitotic catastrophe.	In silico + *in vitro*	Tier 4. As above: intervention is at AKT/FOXO3, with FOXM1 measured downstream. Mitotic catastrophe is an expected consequence of perturbing this axis irrespective of FOXM1 mediation.	(183)
Andrographolide	Indirect	Natural product (diterpenoid)	ROS-dependent downregulation of the FOXM1–ERα module synergizes with fulvestrant in ER+ disease.	*In vitro* + *in vivo*	Tier 4. The proximate intervention is oxidative stress, which alters many transcriptional programs. FOXM1 is co-modulated with ERα, and the two contributions were not separated.	(184)
Mistletoe extract	Indirect	Natural product (extract)	Targets the STAT3–FOXM1 pathway by reducing RAD51 and survivin, inducing apoptosis, and suppressing metastasis.	*In vitro* + *in vivo*	Tier 4. An undefined multi-component extract acting on STAT3, itself a broad regulator of survival and repair genes. Attribution to FOXM1 is not supported by any FOXM1-specific control.	(185)
Dinaciclib	Indirect	Small molecule (CDK1/2/5/9 inhibitor)	It lowers FoxM1- and Hedgehog-associated stemness markers (OCT4, NANOG, SOX2), reducing self-renewal.	*In vitro*	Tier 4. A multi-CDK inhibitor. CDK9 inhibition suppresses transcription broadly, so reduction of FOXM1 and of stemness markers is expected without FOXM1 mediation.	(186)
Gefitinib	Indirect	Small molecule (EGFR inhibitor)	Represses FOXM1 via FOXO3a in sensitive cells. Note: This study carries an Expression of Concern and should be interpreted cautiously.	*In vitro* + clinical samples	Not tiered. Excluded from the synthesis: this study carries an Expression of Concern regarding data integrity. Retained as a flagged note only; no claim in this review depends on it.	(187)
Targeted delivery, aptamers and nanomedicine						
FOXM1 aptamer + DOX (TiO_2_ NP)	Delivery	Nucleolin-targeted pH-responsive nanoparticle	Co-delivers FOXM1 aptamer and doxorubicin with selective uptake; inhibits tumor growth 1.4–1.7× more than free or non-targeted drugs.	*In vitro* + *in vivo*	Tier 2. Cargo specificity is high by design, but efficacy arises from a multi-component system co-delivering doxorubicin; the FOXM1-attributable fraction of the antitumor effect was not isolated.	(188)
Liposomal FOXM1 aptamer	Delivery	Liposome	Reduces doxorubicin resistance, increases Dox cytotoxicity and apoptosis, and improves antitumor efficacy over Dox alone.	*In vitro* + *in vivo*	Tier 2. The comparison against doxorubicin alone supports a contribution from the aptamer arm, but aptamer engagement of FOXM1 was not independently verified and no scrambled-aptamer control is reported.	(189)
AuNP + AS1411/ATP + FOXM1 aptamer + DOX	Delivery	Gold nanoparticle (multi-aptamer)	Nucleolin-targeted co-delivery of FOXM1 aptamer and doxorubicin reduces tumor growth relative to free doxorubicin with lower off-target toxicity.	*In vitro* + *in vivo*	Tier 2. Three functional elements (AS1411, ATP aptamer, FOXM1 aptamer) plus a cytotoxic payload. The FOXM1-specific contribution cannot be separated from targeting and delivery effects.	(190)
FOXM1 siRNA + TGF-β1 siRNA NP	Delivery	Polymeric nanoparticle (PEG-PDMAEMA)	Co-silences FOXM1 and TGF-β1 with synergistic suppression of viability, migration, invasion, and EMT; targets TNBC and bone metastasis.	*In vitro*	Tier 2. siRNA affords sequence-defined target specificity, the strongest reagent specificity in this section, but the reported effect is explicitly synergistic with TGF-β1 silencing, so the FOXM1 arm is not evaluated in isolation.	(191)
DNA nanostructure + FOXM1/AS1411 aptamer + epirubicin + antimiR-10b	Delivery	Linear DNA nanostructure	Nucleolin-targeted co-delivery of epirubicin and antimiR-10b; greater tumor inhibition than free epirubicin	*In vitro* + *in vivo*	Not attributable to FOXM1. In this construct the FOXM1 aptamer functions as a structural and targeting element; the reported antitumor activity is attributed by the authors to epirubicin and antimiR-10b.	(192)

Tiers grade the evidence for attributing an observed effect to FOXM1, not therapeutic promise, and are defined in the preamble to Section 12. Tier 1: direct binding, selective transcriptional consequence, and epistasis or rescue evidence. Tier 2: designed against or reported to engage FOXM1, lacking one or more of these. Tier 3: engages FOXM1 but carries independently documented activity sufficient to produce the same phenotype. Tier 4: lowers FOXM1 downstream of an upstream perturbation, FOXM1-specific contribution not isolated. No agent in this table has a validated pharmacodynamic biomarker of *in vivo* target engagement.

### 12.1 Direct small-molecule inhibitors and repurposing

Efforts to engage FOXM1 pharmacologically have produced an unusually heterogeneous chemical landscape; however, the studies assembled here suggest that the more informative axis of comparison is not chemical class but the mechanism by which FOXM1 activity is reduced. Across this work, three broad modes of engagement recur: direct suppression of FOXM1 transcriptional activity, induction of FOXM1 protein degradation, and modulation of the wider pathway in which FOXM1 operates, each arrived at through a distinct discovery philosophy. These approaches appear complementary rather than hierarchical, and no single strategy is established as superior.

Direct inhibition is the most frequently represented mode, and it is also where the strongest target-engagement evidence in this field is found. The tool inhibitor FDI-6, identified in a high-throughput screen of 54,211 compounds as a blocker of FOXM1 DNA binding, was shown to bind FOXM1 protein directly, to displace FOXM1 from its genomic targets in MCF-7 breast cancer cells, and, by RNA sequencing, to downregulate FOXM1-activated genes selectively, with FOXM1 occupancy at those genes confirmed by ChIP-PCR (170). This combination of direct binding, chromatin displacement and transcriptional selectivity, established in a breast cancer background, is the most complete validation package available for any FOXM1-directed small molecule and is the standard against which the remaining agents are assessed here (Tier 1). In subsequent application to triple-negative models, FDI-6 downregulated FOXM1 together with CyclinB1, Snail and Slug, and suppressed proliferation, migration and invasion with increased apoptosis (171); a structure-guided compound identified by in silico screening has been reported, in conference abstract form only, to suppress FOXM1 with greater potency than that reference inhibitor (172);consistent with the criteria set out in Section 3, this is noted for completeness and is not counted as independent evidence, and a later inhibitor generation achieves substantially greater efficiency in lowering both endogenous and therapy-induced FOXM1 activity (173). A mechanistically distinct route promotes loss of FOXM1 rather than blockade of its activity, through compound-induced relocalization of nuclear FOXM1 to the cytoplasm and subsequent autophagic degradation (78). Notably, suppression of activity and elimination of protein converge on the same phenotypic readout, reduced FOXM1-dependent transcription, while differing in whether the target is the functioning protein or its abundance.

The discovery platforms themselves illustrate complementary logics. Repurposing of approved drugs identified proton-pump inhibitors as unexpected FOXM1 binders (174), network-centric transcriptomic analysis nominated a selective degradation-inducing compound (78), and connectivity map–based bioinformatics surfaced compounds predicted to reverse a coordinated FOXM1-high/PPARA-low signature (175). In parallel, *de novo* medicinal chemistry and structure–activity work on 2-aminothiazole derivatives targeting the FOXM1 DNA-binding domain yielded candidates that outperformed the reference inhibitor *in vitro* (176). Repurposing offers speed and known pharmacology, whereas rational design offers control over selectivity and target geometry; the evidence supports their coexistence rather than a ranking.

The recurring theme is the combinability of these approaches. FOXM1 inhibition has been shown to have synergistic or additive interactions with proteasome and CDK4/6 inhibitors across breast cancer subtypes (169). Degradation-based suppression sensitizes cells to platinum agents, 5-fluorouracil, and taxanes (78), and activity-based inhibition broadly increases sensitivity to conventional therapies, an effect lost in FOXM1-depleted cells and, therefore, attributable to FOXM1 suppression (173). This last design deserves emphasis, since it is the only instance in the material reviewed here in which a compound’s phenotype was shown to require FOXM1: the sensitising effect was abolished in cells already depleted of FOXM1, which excludes an FOXM1-independent route to the same endpoint. Reports lacking this control establish that an agent lowers FOXM1 and that a phenotype follows, but not that the second depends on the first.These observations are consistent with FOXM1-directed agents acting as combination partners, although synergy is demonstrated only in specific pairings and is not universal.

Collectively, these findings suggest that the engagement mechanism, rather than the scaffold, organizes therapeutic thinking about FOXM1. A specific attribution problem qualifies one widely used agent and, with it, part of the argument developed earlier in this review. Thiostrepton interacts directly with FOXM1 and inhibits its binding to genomic targets (59), but thiostrepton and siomycin A also inhibit the proteasome, while structurally unrelated proteasome inhibitors such as MG115, MG132, and bortezomib likewise suppress FOXM1 activity and expression (60). Thus, reduction of FOXM1 by thiostrepton cannot by itself establish selective FOXM1 dependence, and we classify thiostrepton as Tier 3. The same consideration applies to combination data: FOXM1 inhibitors show enhanced activity when combined with proteasome inhibitors (169), but because proteasome inhibition itself can suppress FOXM1 (60), such synergy does not by itself establish cooperation between FOXM1 inhibition and an orthogonal mechanism. More broadly, the agents considered here remain preclinical, and the relationship between FOXM1 target engagement and phenotypic response remains incompletely defined. Comparative superiority among the different modalities has likewise not been established.

### 12.2 Peptides, degraders and condensate disruption

Beyond small-molecule inhibition of FOXM1 transcriptional activity, a second wave of approaches engages FOXM1 through physically distinct mechanisms that occupy progressively different levels of biological organization, from interference with protein–protein contacts, through induced degradation, to disruption of higher-order molecular assemblies. Within the limits of the current data, these modalities appear complementary rather than hierarchical, and none is established as superior. At the level of function, an interfering peptide binds the C-terminal region of FOXM1 and disrupts its interactions with PLK1 and CBP while impeding nuclear entry, thereby suppressing transcriptional activity without removing the protein (177). Degradation strategies pursue the alternative goal of eliminating the protein itself: a peptide-based PROTAC linking a FOXM1-binding antagonist peptide to an E3-ligase ligand induces FOXM1 degradation (178), while EZH2-directed PROTACs degrade an EZH2–FOXM1-associated node, lowering shared target-gene expression (179). Notably, these illustrate a conceptual distinction between single-target elimination and disruption of a multi-protein oncogenic node. The downstream readouts overlap, reduced proliferation, migration, tumor growth, and metastasis, across interference and degradation alike; the reported decreases in GLUT1 and PD-L1 accompanying FOXM1 degradation (178) are downstream observations within that study rather than independent evidence of metabolic or immune reprogramming.

A third, biophysically distinct mode targets FOXM1 liquid–liquid phase separation: AMPK-mediated phosphorylation within the intrinsically disordered region, or a peptide carrying the phosphorylated residue, perturbs FOXM1 condensates and reduces oncogenic transcription (6) (single study, not independently replicated). Perturbing higher-order organization differs conceptually from reducing protein abundance or blocking a binding interface, and these approaches should not be considered interchangeable. One therapeutic implication of Section 3.4 is that preventing condensate formation and disrupting an established condensate may not be biophysically equivalent. In model systems, condensates can undergo time-dependent changes in material state (68, 69), and less dynamic intracellular assemblies can disassemble more slowly and progressively lose reversibility (67). If FOXM1 condensates undergo similar ageing in tumor cells, the response to condensate-disrupting agents could depend on condensate age or material state, making treatment timing a potential pharmacodynamic variable. This has not been tested for FOXM1. The available study demonstrates disruption of FOXM1 LLPS with suppression of tumor growth and metastasis (6), but does not assess whether efficacy varies with condensate age or prior duration of FOXM1 elevation. Thus, material-state dependence should be considered a testable issue in the development of condensate-directed therapies (180, 181), rather than an established limitation of current data.

Together, these modalities indicate that FOXM1-associated functions can be perturbed at multiple levels: functional interference, protein degradation, node-centered degradation, and disruption of higher-order assemblies. However, these modalities remain largely preclinical, comparative superiority is unknown, validated pharmacodynamic biomarkers of on-target engagement are lacking, the selectivity and mechanistic basis of condensate disruption are incompletely established, and the relationship between target engagement and phenotypic response remains unresolved; these considerations motivate the indirect and delivery-based strategies discussed next.

### 12.3 Indirect and natural-product-mediated suppression

Not every approach to constraining FOXM1 requires the physical engagement of the protein. A distinct therapeutic space is occupied by natural products and signaling-targeted agents that lower FOXM1 expression or activity as a downstream consequence of perturbing the regulatory circuits that sustain it. Conceptually, these strategies differ from direct inhibition, degradation, or condensate disruption: they act on upstream dependencies rather than on FOXM1 itself, and within the limits of the current data, it cannot be assumed that they are equivalent or superior to direct modalities. The studies reviewed here converge on several regulatory entry points. A recurring axis is AKT/FOXO3, where suppression of AKT signaling restores FOXO3 and lowers FOXM1, observed both with a flavone in an Akt-driven endocrine-resistant context (182) and with an alkaloid that drives mitotic catastrophe through the AKT/FOXO3/FOXM1 axis with concomitant PLK1 reduction (183). A parallel ROS-dependent route downregulates a FOXM1–ERα module and synergizes with fulvestrant (184), while STAT3 inhibition coincides with reduced FOXM1 alongside RAD51 and survivin (185), and CDK inhibition lowers FOXM1- and Hedgehog-associated stemness markers (186). One further report, linking EGFR inhibition to FOXM1 repression via FOXO3a (187), is excluded from this synthesis because it carries an Expression of Concern regarding data integrity; it is noted here for completeness only, and no claim in this review depends on it.

These interventions produce overlapping phenotypes, including reduced proliferation and tumor growth, apoptosis, enhanced endocrine therapy efficacy, reduced metastatic behavior and stemness, and mitotic catastrophe. However, FOXM1 attribution remains unresolved. The interventions perturb upstream signaling through AKT/FOXO3 (182, 183), ROS/ERα (184), STAT3 (185), or CDK/Hedgehog pathways (186), with FOXM1 modulation occurring within these responses. Because these pathways also regulate the observed phenotypes through effectors other than FOXM1, the available data do not establish FOXM1 as the principal mediator. In the absence of definitive FOXM1-dependence or rescue experiments, these agents are therefore classified as Tier 4: their effects show that FOXM1 is modulated by pathway perturbation, but do not establish that the phenotype is mediated predominantly through FOXM1.

These findings indicate that FOXM1-associated programs can be indirectly constrained by targeting their upstream regulatory dependencies, an intervention at a different level of the FOXM1 hierarchy than direct engagement. However, most evidence is preclinical, the relative efficacy of indirect versus direct strategies is unknown, multi-pathway activity makes FOXM1-specific contributions difficult to isolate, biomarkers of selective pathway engagement are insufficiently defined, and subtype context dependency remains incompletely understood. These constraints motivate the development of emerging strategies aimed at improving targeting precision and implementation.

### 12.4 Targeted delivery, aptamers and nanomedicine

A further dimension of FOXM1 therapeutics is defined not by a new mode of molecular antagonism but by control over where, how, and in what molecular company a FOXM1-directed agent reaches tumor cells. Here, the distinction between vehicle and cargo becomes central: the inhibitory payload may be a FOXM1 aptamer or FOXM1 siRNA, while engineered architectures, lipids, polymers, metals, and nucleic acid-based systems determine its delivery and cellular targeting. These platforms operate at the level of deployment rather than of FOXM1 biology itself. A recurring design principle is combinatorial co-delivery. FOXM1-targeting cargos are repeatedly paired with anthracycline chemotherapeutics, co-delivery of a FOXM1 aptamer with doxorubicin (188), a liposomal FOXM1 aptamer used to reduce doxorubicin resistance (189), gold-nanoparticle systems carrying both doxorubicin and a therapeutic FOXM1 aptamer (190), or with additional regulatory oligonucleotides, as in co-loading of FOXM1 siRNA with TGF-β1 siRNA (191) or a DNA nanostructure pairing a FOXM1 aptamer with epirubicin and antimiR-10b (192). Tumor-cell selectivity is frequently conferred by an independent targeting element, commonly the nucleolin-binding AS1411 aptamer (188, 190, 192). Notably, because efficacy arises from integrated multi-component systems, the reported outcomes should not be attributed to FOXM1 modulation alone.

These constructs are associated with overlapping outputs, suppressed breast cancer growth, improved responses in doxorubicin-resistant contexts, and activity in triple-negative and bone-metastasis models, with synergy reported for paired-siRNA EMT modulation (191). Two comparisons with the wider TNBC nanomedicine field help to situate these constructs. First, nanoplatforms are increasingly designed to modulate the tumor microenvironment and antitumor immunity rather than a single transcriptional target. A recent pH-responsive, CXCR4-targeted platform co-assembling the PARP inhibitor veliparib with a Mn²^+^ coordination core enhanced antitumor immune responses in TNBC through mechanotransduction (193). This platform is not FOXM1-directed and is cited for context rather than as evidence for FOXM1. Second, it illustrates a distinction shared by the FOXM1-directed systems above: nanoformulation can alter drug delivery and tumor exposure but does not, by itself, prevent transcriptional adaptations underlying resistance. In the PARP-inhibitor setting, olaparib induces FOXM1 and DNA-repair programs, whereas FOXM1 or PI3K inhibition increases olaparib sensitivity in BRCA-proficient TNBC models (161, 162). Thus, optimized PARP-inhibitor delivery and FOXM1-directed intervention address different components of treatment failure and may be complementary rather than alternative strategies. This remains a framework-based prediction, however, as the combination of FOXM1-directed therapy with an optimized PARP-inhibitor delivery platform has not been established. Viewed together, these delivery studies show a layered engineering logic: cargo identity, additional payloads, targeting ligands, and multifunctional architecture. Conceptually, this contrasts with direct inhibition, degradation, condensate disruption, or indirect pathway suppression, which alter FOXM1 function or abundance, whereas delivery design alters the context of deployment; these are not interchangeable.

However, the evidence is largely preclinical; comparative superiority among architectures is unknown; the specific contribution of FOXM1 suppression within multifunctional systems has not been isolated in any of these reports, which is the basis for their evidence-tier assignment in **Table 4**; whether enhanced experimental efficacy translates to broader benefit is unresolved; and biomarkers linking delivery efficiency to FOXM1-pathway suppression remain insufficiently defined. A further distinction should be kept in view: delivery engineering and mechanism-directed intervention address different failure modes, the first insufficient drug exposure and the second adaptive transcriptional resistance, and improvements in one do not substitute for the other. These attribution challenges frame the broader integration of FOXM1-targeting modalities, which will be discussed next.

## 13 Limitations, research gaps, and future perspectives

Despite a coherent organizing framework, the evidence assembled here carries intrinsic limitations. The prognostic association of FOXM1 remains correlative rather than causal (21, 22), and its functional “activity” is inferred almost entirely from bulk expression, leaving FOXM1 dose without a quantitative, isoform-resolved definition (22). Consequently, the central claim that a single dosage set-point selects discrete output states is at present a synthesis hypothesis rather than a demonstrated mechanism. At each regulatory tier, hierarchy is unresolved. A further variable is absent rather than unresolved. YAP1 has been implicated in the regulation of FOXM1 in breast cancer (99), while FOXM1 has also been shown to promote breast cancer stemness and migration in a YAP1-dependent manner (146). Because YAP1 and TAZ are major mediators of mechanotransduction, integrating extracellular-matrix rigidity, cell shape and cytoskeletal tension into transcriptional responses (150), the mechanical state of the cellular microenvironment is a plausible determinant of the FOXM1 set-point. Yet the evidence assembled here does not directly establish how mechanical inputs affect FOXM1 output. The titration experiments proposed above should therefore be repeated across defined substrate stiffnesses and, where feasible, controlled matrix composition and cytoskeletal tension, because a FOXM1 dose–response relationship established under conventional culture conditions cannot be assumed to hold across different mechanical microenvironments. Whether genomic, enhancer, and transcription factor inputs act hierarchically, additively, or context-specifically is unknown, and predicted FOXM1 variants await experimental validation (101). Post-transcriptionally, the relative weight of miRNA, ceRNA, and m6A inputs is unquantified, and feed-forward loops obscure cause from consequence (104). Post-translationally, the stoichiometry and ranking among competing deubiquitinases and cross-talk between the ubiquitin, phosphorylation, and glycosylation layers remain unexamined (49, 74–76, 79–84). A specific correction bears on this tier. One reported FOXM1 deubiquitinase, USP39, is a catalytically inactive pseudo-DUB (111, 112), so the mechanism by which it lowers FOXM1 ubiquitination is unresolved and may not be post-translational at all. Because USP39 is a spliceosome assembly factor (114), the alternative that it acts on FOXM1 isoform composition at Tier II should be tested directly, by junction-resolved isoform quantification and half-life measurement after USP39 depletion in the same cells. The same requirement applies generally: where an enzyme’s assignment to this tier rests on a reported ubiquitination readout alone, catalytic competence toward FOXM1 should be demonstrated rather than inferred from family membership.

The decisive gap for the output states and resistance convergence is resolution. Whether mitotic, metabolic, immune, and stem-like programs are co-regulated within individual cells, and whether mechanistically distinct resistance phenotypes constitute one molecular state, cannot be determined from bulk, cross-sectional data; genome rewiring documents plasticity without establishing a shared attractor (65). Establishing an attractor in the dynamical sense, the convergence of mechanistically distinct resistance trajectories onto a common, stable transcriptional state, requires single-cell trajectory inference (pseudotime, RNA velocity, or heritable lineage tracing), which is currently lacking (16, 66). The reversibility of the plastic state has not been directly tested (151). Two sources warrant explicit handling and are treated differently. One report bearing on the indirect-therapeutic section carries an Expression of Concern regarding data integrity (187) it has been excluded from the synthesis, is retained only as a flagged note, and supports no conclusion here. Separately, the innate-immune (STING) arm of the immune-evasion state rests on a single peer-reviewed study together with a closely matching conference abstract of the same work (143), this is a single, not yet independently replicated line of evidence rather than a data-integrity concern, and the immune-evasion synthesis does not depend on it, being additionally supported by the macrophage-education and checkpoint arms. Future work should therefore prioritize single-cell, dose-resolved multi-omics across titrated FOXM1 gradients, designed specifically to adjudicate between the state-selector and monotonic-hub models. Three signatures would distinguish them: threshold nonlinearity, in which output programs engage at discrete FOXM1 levels rather than scaling continuously with dose; multimodality with partial mutual exclusivity, in which single cells partition into discrete output states rather than forming a unimodal continuum; and hysteresis, in which upward and downward titration of FOXM1 yield non-coincident switching thresholds. Observing instead graded, unimodal, path-independent scaling of all outputs would favor the hub null and constrain the present thesis accordingly. These readouts should be complemented by lineage tracing of state transitions and by an isoform-aware activity metric of the kind set out in Section 3.2, in which measured protein abundance is combined with the active-isoform, nuclear and de-repressed fractions and calibrated against an independent output anchor such as the FOXM1 cistrome or its target-gene program (23, 26). Constructing such a metric requires two measurements that are currently absent from the breast cancer literature: junction-resolved isoform quantification in breast tumors, which would also resolve the conflicting isoform hierarchies reported to date (35, 38), and a phosphosite occupancy measurement validated as a proxy for FOXM1 transcriptional activity. The metric is itself falsifiable: should it fail to predict FOXM1-associated output better than total abundance alone, the compositional component of the dosage thesis would be refuted and “dose” would reduce to abundance. These developments should proceed alongside validated pharmacodynamic biomarkers of on-target engagement, in order to advance the largely preclinical therapeutic landscape (78, 171–173, 175, 191, 192, 194) toward clinical translation. The corresponding experimental design can be specified concretely. A single breast cancer background, ideally including one ER-positive and one basal-like/TNBC model, should be engineered for quantitative and reversible control of endogenous FOXM1 abundance, for example by graded transcriptional repression or degron-mediated depletion, allowing intermediate FOXM1 set-points to be tested rather than binary perturbations (71, 72). FOXM1 protein abundance should be quantified at the single-cell level rather than inferred from bulk transcript measurements, with isoform composition measured in parallel where relevant. Each output module should then be measured across at least five FOXM1 levels spanning the relevant range and, where appropriate, fitted with a Hill dose–response function. The resulting half-maximal effective level and slope would provide a quantitative comparison of output sensitivity and test whether mitotic, metabolic, immune-associated and stem-like phenotypes respond within a common FOXM1 dosage range or show distinct sensitivities. To distinguish graded responses from discrete state transitions, these outputs should be measured simultaneously in single cells, particularly at intermediate FOXM1 levels. Hysteresis should be tested by titrating FOXM1 in both increasing and decreasing directions under matched conditions and comparing the corresponding transition points; separated thresholds would support path dependence, whereas overlapping thresholds would provide no evidence for hysteresis under the tested conditions. Finally, because FOXM1 repression has been shown to increase mitotic death during antimitotic chemotherapy through BMF upregulation (63), the titration should be performed both with and without a defined mitotic or therapeutic challenge, allowing FOXM1 dosage effects to be separated from context-dependent effects on mitotic cell fate. Establishing dynamical convergence requires a longitudinal design in which resistant populations are generated in parallel from a common parental population under distinct therapeutic pressures, with single-cell profiling at multiple stages and a shared analytical reference. Heritable lineage barcoding can further distinguish the contribution of pre-existing lineages and determine whether similar resistant states arise from the same or different cellular ancestries. This approach is supported by a barcoded persister system showing that cycling and non-cycling persisters arise from distinct lineages with distinct transcriptional and metabolic programs across multiple cancer models (153), illustrating how lineage and state can diverge under drug pressure.

The titration design described above also permits a direct test of the phase-boundary formulation proposed in Section 3.3, and the two should be run together rather than separately. Because reversible control of FOXM1 abundance generates a per-cell concentration series, the saturation concentration can be determined in the same experiment by relating the appearance of condensates to total FOXM1 concentration cell by cell, using quantitative fluorescence imaging of a tagged endogenous allele. Three questions follow. Whether the switching level for each output program coincides with the measured saturation concentration, which the phase-boundary model requires and the abundance-only model does not. Whether, above that concentration, target-gene output tracks condensate number and volume fraction rather than nucleoplasmic FOXM1, which distinguishes partitioned amount from free concentration as the operative analog variable. And whether manipulating the disordered region, by mutating the AMPK phosphosite or by AMPK activation (6), shifts the measured saturation concentration and shifts the output threshold with it, which would establish post-translational modification as a tuner of the phase boundary rather than only of abundance. Because heterotypic interactions dominate intracellular phase separation (86), the saturation concentration should be measured *in situ* rather than inferred from reconstituted material, and cofactor availability should be recorded alongside it.

The SQLE association identified in Section 8 can be tested using four complementary approaches. First, glycolysis should be quantified by glycolytic proton production rate corrected for medium buffering and respiration-derived acidification (129, 130), together with ¹³C_6_-glucose tracing into lactate and TCA-cycle intermediates to assess glucose-carbon partitioning; isotope labeling alone, however, should not be interpreted as quantitative flux without appropriate flux analysis (195). Second, SQLE dependence should be evaluated by SQLE knockdown and pharmacological inhibition, complementing the FOXM1-knockdown/SQLE-rescue experiments already reported by Xu et al.; attenuation of the glycolytic phenotype would support, but not prove, SQLE dependence (125, 128, 139). Third, cholesterol rescue, combined with measurement and supplementation of squalene, could distinguish reduced downstream sterol availability from squalene accumulation. In K-ras-driven pancreatic cancer, SQLE inhibition was not cholesterol-rescuable and was associated with squalene accumulation (137), whereas squalene accumulation protected cholesterol-auxotrophic lymphoma cells from oxidative cell death (138), demonstrating context-dependent effects. Fourth, upstream regulation should be examined because mTORC1 regulates glycolytic and lipid-biosynthetic programs through HIF1α- and SREBP-associated pathways (136); therefore, co-suppression of SQLE and glycolytic readouts after mTORC1 inhibition would be consistent with, but would not establish, shared regulation, while selective SREBP perturbation could test their dissociation. Finally, measurements should be performed under both attached and detached conditions because Xu et al. studied anoikis using ultra-low-attachment culture but measured ECAR under conventional Seahorse conditions (125), whereas matrix detachment itself can reduce glucose uptake and ATP and increase oxidative stress, with pentose phosphate pathway activity contributing to antioxidant protection (196).

Trajectory inference can then be performed using RNA velocity (197) or optimal-transport approaches for time-resolved single-cell data (198). These methods remain inferential and depend on modeling assumptions; RNA velocity, in particular, has documented limitations and failure modes (199). Trajectory predictions should therefore be integrated with lineage information rather than interpreted as direct measurements of cell fate. Testing the material-state formulation of Section 3.4 requires three additions to the same experimental design. First, hold time should be treated as an independent variable by initiating the descending arm after defined periods at the same elevated FOXM1 level. A hold-time-dependent shift in the switch-off threshold would indicate involvement of a slowly evolving state variable, potentially including condensate ageing, but would not by itself distinguish ageing from slow feedback, protein turnover, post-translational modification or chromatin remodeling.

Second, condensate dynamics should be measured with age resolution, comparing newly formed and longer-lived assemblies within the same cells. FRAP provides an accessible measure of molecular mobility and exchange, but its interpretation depends on bleaching geometry and the fitting model, which should therefore be reported explicitly (200).

Third, condensate behavior and FOXM1 target-gene output should be paired at the single-cell level, because persistence of an assembly does not necessarily imply persistent transcriptional activity (180, 201). In-cell measurements should be prioritized because heterotypic interactions and chromatin mechanics can strongly influence intracellular phase behavior and condensate dynamics (86, 92, 93). If endogenous condensates are too small for reliable analysis, light-inducible systems can be used to examine phase boundaries and nucleation behavior (67, 202), while recognizing that engineered constructs demonstrate physical plausibility rather than endogenous FOXM1 behavior.

Two functional tests would further distinguish a stable resistant state from a drug-maintained response. First, resistant populations should be examined after drug withdrawal to determine whether the resistant configuration persists or returns toward the parental state (153). Second, reciprocal drug sensitivity can test whether independently derived resistant populations share functional vulnerabilities. FOXM1-inhibitor- and CDK4/6-inhibitor-resistant breast cancer cells provide a relevant example: despite shared JAK/STAT1–interferon–ISGylation features, their differential drug sensitivities permit reciprocal sequential targeting (165). Extending this strategy across endocrine, DNA-damage and antimitotic therapies would provide a direct functional test of whether apparently convergent resistance programs represent a shared state or distinct configurations.

## 14 Conclusion and outlook

The framework advanced here proposes repositioning FOXM1 from a conventional proliferation-associated oncogene to a dosage-integrating regulator whose set-point is established by layered genomic, epitranscriptomic and post-translational inputs and translated into a repertoire of co-occurring outputs: mitotic and chromosomally unstable, metabolic, immune-evasive, and stem-like. Whether those outputs constitute discrete states in the sense the model requires is the open question of this article, not its conclusion. The observations that motivate this framing are convergences: structurally unrelated perturbations contract multiple programs in parallel, subtype-divergent upstream inputs, endocrine in ER+ disease and proliferation/E2F-network-driven in basal-like/TNBC disease, feed a shared FOXM1-associated output, while the corresponding evidence in HER2-amplified disease is absent rather than negative (**Table 2**), and mechanistically distinct therapeutic pressures directed elsewhere in the network are repeatedly buffered by the persistence or reactivation of that output rather than by separate effectors, although resistance to FOXM1 inhibition itself proceeds by an alternative route, indicating that the FOXM1-associated configuration is one accessible resistance state among several rather than a universal one. Each of these is a convergence, and convergence alone, as emphasized in Section 3, establishes FOXM1 as a shared node without discriminating the rheostat model from a linear multi-output hub. Viewed this way, FOXM1 is most usefully understood not as a linear driver but as a shared node at which adaptive plasticity and therapy resistance may be organized, and as a candidate point of pharmacological convergence approachable through inhibition, degradation, condensate disruption, indirect pathway suppression, and targeted delivery (60, 170). Two qualifications attach even to that weaker statement. The agents currently available to test the proposition are, with limited exceptions, not validated as FOXM1-selective, and resistance to direct FOXM1 inhibition may proceed through reversal rather than persistence of FOXM1-associated output (65), indicating that the configuration described here is one accessible resistance state among several rather than a universal one.

However, this interpretation remains a hypothesis rather than an established mechanism. The evidence is largely correlative, derived from bulk measurements, and devoid of the single-cell, dose-resolved, and isoform-aware data required to demonstrate that FOXM1 dose actively selects discrete cellular states. Therefore, the field’s central task is to convert a compeling organizing principle into a testable, quantitative model through titrated perturbation across single-cell multi-omic readouts, lineage tracing of state transitions, and validated pharmacodynamic biomarkers of on-target engagement. Resolving whether FOXM1 functions as a true state selector, evidenced by threshold-structured, partly mutually exclusive, hysteretic output states at single-cell resolution, or merely as a highly connected hub whose outputs scale smoothly and monotonically with dose will determine whether its therapeutic targeting can be rationally matched to dependency, and ultimately translated from preclinical promise into durable clinical benefit.
